# Deletion of neurosecretory proteins GL and GM drives dual anti-obesity effects via appetite suppression and enhanced energy expenditure

**DOI:** 10.1038/s42003-026-10405-7

**Published:** 2026-06-03

**Authors:** Kenshiro Shikano, Mitsuhiro Yoshimura, Takaki Yahiro, Taishi Kurakazu, Shigeyoshi Saito, Hitoshi Teranishi, Ryoko Higa, Ryohei Umeda, Magdeline E. Carrasco Apolinario, Takashi Maruyama, Atsuo T. Sasaki, Toshikatsu Hanada, Takatoshi Hikida, Kazuyoshi Ukena, Josef M. Penninger, Yoichi Ueta, Chikashi Terao, Kazuhiro Nakamura, Reiko Hanada

**Affiliations:** 1 Department of Physiology, Faculty of Medicine, Oita University, Yufu, Oita Japan; 2 Department of Physiology, School of Medicine, University of Occupational and Environmental Health, Kitakyushu, Japan; 3 Department of Integrative Physiology, Nagoya University Graduate School of Medicine, Nagoya, Japan; 4 Vollum Institute, Oregon Health and Science University, Portland, OR USA; 5 Laboratory for Statistical and Translational Genetics, RIKEN Center for Integrative Medical Sciences, Yokohama, Kanagawa Japan; 6 Department of Medical Physics and Engineering, Division of Health Sciences, The University of Osaka Graduate School of Medicine, Suita, Osaka Japan; 7 Department of Medicine, New York University Grossman School of Medicine, New York, NY USA; 8 Department of Advanced Medical Science, Faculty of Medicine, Oita University, Oita, Japan; 9 Department of Internal Medicine, University of Cincinnati College of Medicine, Cincinnati, USA; 10 Institute for Advanced Biosciences, Keio University, Tsuruoka, Japan; 11 Department of Clinical and Molecular Genetics, Hiroshima University, Hiroshima, Japan; 12 Department of Biochemistry and Molecular Genetics, Faculty of Medicine, Oita University, Yufu, Oita Japan; 13 Laboratory for Advanced Brain Functions, Institute for Protein Research, Osaka University, Osaka, Japan; 14 Laboratory of Neurometabolism, Graduate School of Integrated Sciences for Life, Hiroshima University, Higashi-Hiroshima, Hiroshima Japan; 15 Institute of Molecular Biotechnology of the Austrian Academy of Sciences, Vienna, Austria; 16 Department of Medical Genetics, Life Sciences Institute, University of British Columbia, Vancouver, BC Canada; 17 Helmholtz Centre for Infection Research, Braunschweig, Germany; 18 Department of Laboratory Medicine, Medical University of Vienna, Vienna, Austria; 19 Clinical Research Center, Shizuoka General Hospital, Shizuoka, Japan; 20 The Department of Applied Genetics, The School of Pharmaceutical Sciences, University of Shizuoka, Shizuoka, Japan; 21 Department of Statistical Genetics, Center for Genomic Medicine, Research Promotion Headquarters, Fujita Health University, Toyoake, Aichi Japan

**Keywords:** Obesity, Obesity

## Abstract

Obesity results from an imbalance between energy intake and expenditure and is regulated by hypothalamic neuropeptide systems. The neurosecretory proteins GL (NPGL) and GM (NPGM) are expressed in the hypothalamus and promote feeding in gain-of-function studies; however, their endogenous physiological roles remain unclear. Here, we show that mice lacking both NPGL and NPGM display a lean phenotype driven by reduced food intake and increased energy expenditure. This anti-obesity phenotype is associated with increased expression of anorexigenic pro-opiomelanocortin in the hypothalamus and enhanced thermogenic activity in brown adipose tissue, marked by elevated uncoupling protein 1. Consistent with these findings, suppression of NPGL/NPGM signaling reduces feeding and alters sympathetic nerve activity. In addition, genome-wide association analysis identifies an obesity-associated variant near the human NPGM locus, suggesting relevance to human energy balance. Together, these findings identify NPGL and NPGM as endogenous regulators of energy homeostasis with potential relevance to obesity.

**Figure:**
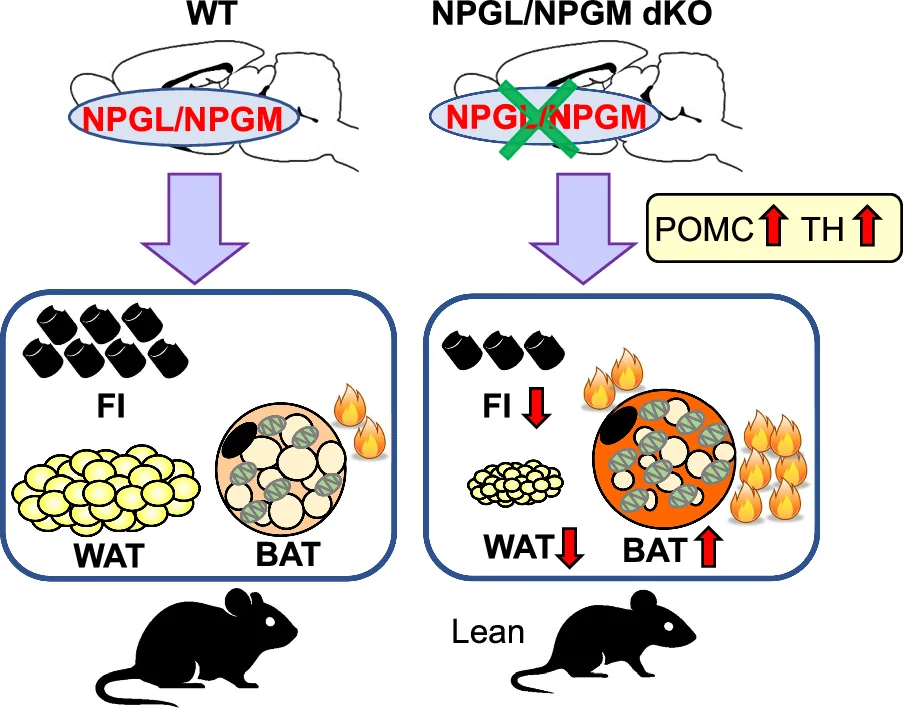

## Abstract (web summary)

Loss of NPGL/NPGM signaling leads to an anti-obesity phenotype mediated by enhanced anorexigenic signaling and increased sympathetic activation of brown adipose tissue. GWAS analysis identifies an obesity-associated variant near the human NPGM locus.

## Introduction

Obesity is a major global health burden and a leading risk factor for metabolic disorders, including type 2 diabetes, cardiovascular disease, and certain cancers^1–4^. At its core, obesity arises from a chronic imbalance between energy intake and energy expenditure. The maintenance of energy homeostasis is therefore essential for metabolic health and is regulated by complex interactions between peripheral metabolic signals and central neural circuits^5,6^.

The hypothalamus plays a central role in integrating hormonal and nutrient-derived signals from peripheral tissues and coordinating appropriate physiological and behavioral responses^7–9^. Within the hypothalamus, the arcuate nucleus (ARC) is a critical hub for the regulation of feeding behavior and energy balance^5^. The ARC contains distinct populations of neurons that exert opposing effects on appetite. Neuropeptide Y (NPY) and agouti-related peptide (AgRP) neurons promote food intake, whereas pro-opiomelanocortin (POMC), cocaine- and amphetamine-regulated transcript (CART), and neuromedin U neurons suppress feeding^10,11^. The dynamic balance between these orexigenic and anorexigenic pathways enables the brain to adjust energy intake and expenditure in response to nutritional status.

In recent years, neurosecretory protein GL (NPGL) and its homolog neurosecretory protein GM (NPGM) have emerged as additional hypothalamic factors implicated in metabolic regulation^12,13^. NPGL and NPGM are predominantly expressed in the ARC in both avian species and rodents and respond to changes in nutrient availability^12–16^. Experimental studies using central administration of these peptides have shown that NPGL promotes food intake and lipid accumulation, at least in part by suppressing POMC neuron activity^17,18^. NPGM has also been linked to metabolic regulation, although its physiological role remains less well defined^16,19^. Together, these findings suggest that NPGL and NPGM participate in the neural control of energy balance.

However, most existing studies of NPGL and NPGM have relied on gain-of-function approaches, such as peptide administration, transgenic overexpression, or chemogenetic activation. While these strategies have provided valuable insights, they do not fully address the endogenous physiological functions of these peptides under normal or obesogenic conditions. In particular, it remains unclear whether NPGL and NPGM are required for maintaining energy balance and how their absence affects whole-body metabolism. Addressing this gap requires a loss-of-function approach that directly interrogates the contribution of endogenous NPGL and NPGM signaling.

To this end, we generated mice lacking NPGL and NPGM individually and in combination. Initial analyses of single knockout mice revealed minimal effects on body weight and adiposity under standard chow conditions, but resistance to diet-induced obesity when challenged with a high-fat diet. Given that NPGL and NPGM are co-expressed in the same hypothalamic neurons, we further examined the metabolic consequences of combined deletion using NPGL/NPGM double knockout mice.

In this study, we show that loss of NPGL and NPGM results in a robust lean phenotype characterized by reduced food intake and increased energy expenditure. Double knockout mice display enhanced resistance to diet-induced obesity, improved glucose handling, and reduced adiposity. Mechanistically, these effects are associated with increased expression of anorexigenic neuropeptides in the hypothalamus, elevated sympathetic activity, and enhanced uncoupling protein 1–dependent thermogenesis in brown adipose tissue. Complementary pharmacological and gene knockdown experiments support a model in which NPGL and NPGM act centrally to suppress anorexigenic and sympathetic pathways. Finally, analysis of human genomic data identifies an obesity-associated variant near the NPGM (FAM237B) locus, suggesting potential relevance of this system to human energy metabolism. Together, these findings define endogenous NPGL and NPGM signaling as an important regulator of energy homeostasis and provide insight into neural mechanisms linking appetite control and thermogenic energy expenditure.

## Results

### Generation and metabolic characterization of *Npgl* and *Npgm* knockout mice

To investigate the physiological roles of NPGL and NPGM in energy metabolism, we generated *Npgl* and *Npgm* single knockout (KO) mice using CRISPR–Cas9-mediated genome editing (Supplementary Fig. 1). Under normal diet (ND) conditions, neither *Npgl*-KO nor *Npgm*-KO mice exhibited significant differences in body weight, food intake, or adipose and liver tissue weights compared with wild-type (WT) littermates (Supplementary Fig. 2). In contrast, when challenged with a high-fat diet (HFD), both *Npgl*-KO and *Npgm*-KO mice displayed significantly reduced body weight gain and food intake relative to WT controls (Supplementary Fig. 3a, b, g, h). *Npgl*-KO mice showed marked reductions in inguinal white adipose tissue (iWAT), epididymal white adipose tissue (eWAT), brown adipose tissue (BAT), and liver weights (Supplementary Fig. 3c–f), whereas *Npgm*-KO mice exhibited a significant decrease in iWAT mass (Supplementary Fig. 3i–l). These results are consistent with previous gain-of-function studies indicating that NPGL and NPGM promote adiposity, and suggest that loss of either peptide confers partial resistance to HFD-induced weight gain^15–17,20^.

### Combined deletion of NPGL and NPGM induces a robust lean phenotype

**Fig. 1:**
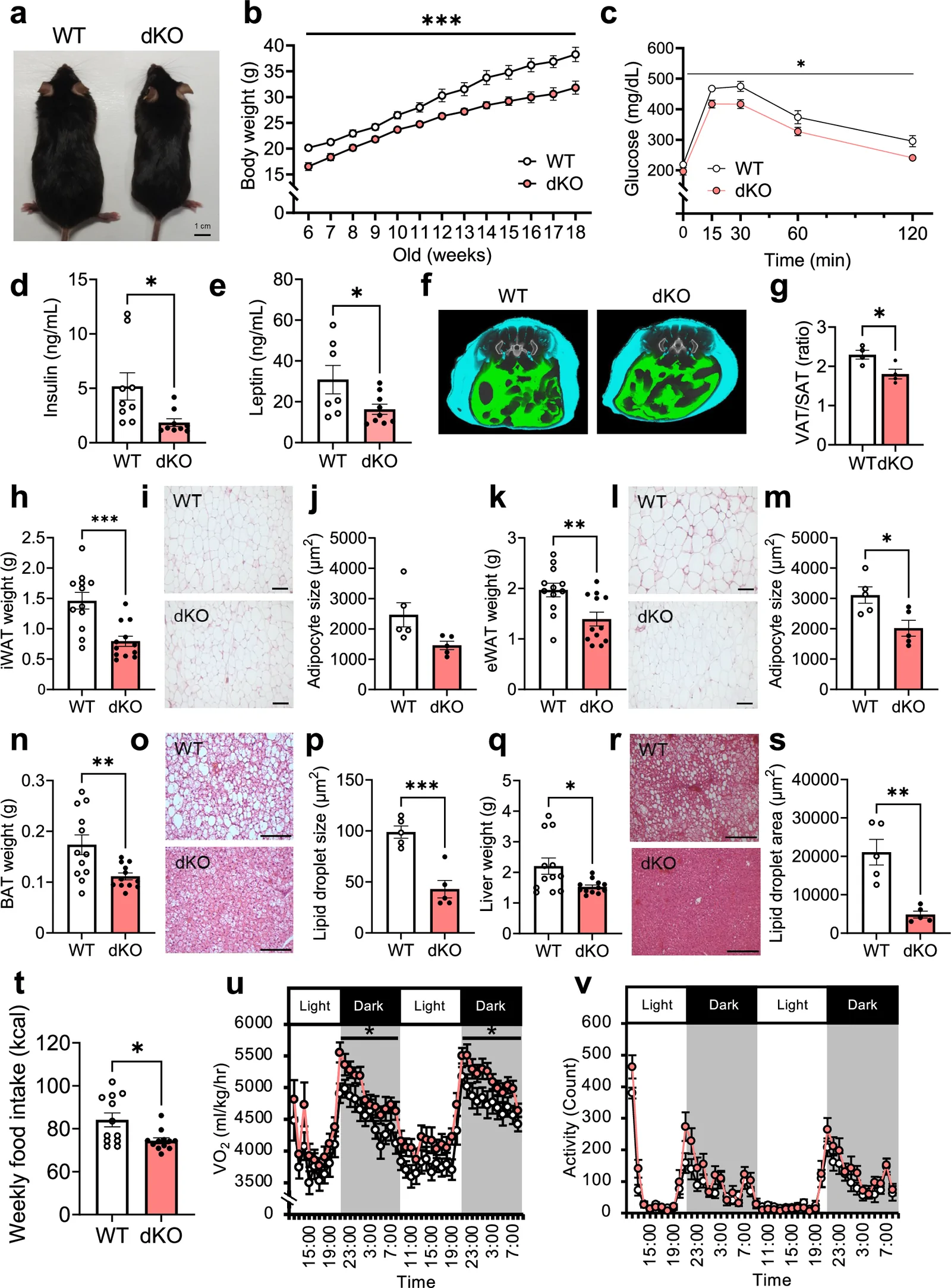
Deletion of both NPGL and NPGM protects against diet-induced obesity. Wild-type (WT) or *Npgl-* and *Npgm*-double knockout (dKO) male mice were fed a high-fat diet (HFD) from 8 weeks of age. **a**, Representative images of WT and dKO mice at 20 weeks of age. **b** Body weight (*n* = 12). **c** Oral glucose test results of mice fed for 16 weeks (n = 15 for WT and *n* = 13 for dKO mice). **d** Serum insulin levels (*n* = 9). **e** Serum leptin levels (*n* = 7 for WT and *n* = 9 for dKO mice). Representative CT images of visceral (green) and subcutaneous (blue) fat in WT and dKO mice at 20 weeks of age (**f**) and Visceral /subcutaneous adipose tissue (VAT/SAT) ratio (**g**) (*n* = 4). Tissue weights (*n* = 12), representative images of HE staining, and adipocyte or lipid droplet size (*n* = 5) of inguinal white adipose tissue (iWAT) (**h**–**j**), epidermal white adipose tissue (eWAT) (**k**–**m**), brown adipose tissue (BAT) (**n**–**p**), and liver (**q**–**s**). **t** Weekly food intake from 12 to 14 weeks of age (*n* = 12). Whole-body oxygen consumption (VO_2_) (**u**) (*n* = 18) and locomotor activity (**v**) in 16-week-old mice (*n* = 16 for WT and *n* = 17 for dKO mice). Data are presented as the mean ± SEM. For bar graphs, each dot represents data from an individual mouse. Asterisks refer to *P*-values obtained from two-way ANOVA adjusted based on Bonferroni’s multiple comparison test (**b**, **c**, **u**, **v**) or an unpaired Student’s *t*-test (**d**, **e**, **g**, **h**, **j**, **k**, **m**, **n**, **p**, **q**, **s**) (*P < 0.05; **P < 0.01; ***P < 0.005). Exact *P*-values are indicated in the Source Data file. Scale bars: 100 μm (**i**, **l**, **o**, **r**).

Given that NPGL and NPGM are co-expressed in the same hypothalamic neurons (Supplementary Fig. 1 f), we next examined the metabolic consequences of their combined deletion. Even under ND conditions, NPGL/NPGM double knockout (dKO) mice exhibited significantly reduced body weight, food intake, and adipose and liver tissue mass compared with WT mice (Supplementary Fig. 4a–m). These differences became more pronounced under HFD conditions (Fig. 1a, b, h–t). Under HFD feeding, NPGL/NPGM-dKO mice showed marked resistance to diet-induced obesity, with lower body weight, reduced adipose and liver mass, and improved glucose tolerance compared with WT controls (Fig. 1a–c, h–s). Although the body weight trajectories of WT and NPGL/NPGM dKO mice appeared largely parallel over time (Fig. 1b), the absolute difference in body weight showed distinct patterns depending on dietary conditions. Under normal chow conditions, there was no significant difference in body weight change between WT and dKO mice from 8 to 18 week-old (WT: 4.41 ± 0.238 g; dKO: 3.92 ± 0.237 g; *P* = 0.156). In contrast, under HFD conditions, dKO mice exhibited significantly reduced body weight gain from 8 to 18 weeks of age compared to WT mice (WT: 15.3 ± 1.01 g; dKO: 11.6 ± 1.03 g; *P* = 0.0187), indicating enhanced resistance to HFD-induced weight gain in NPGL/NPGM dKO mice. These findings suggest that alterations in energy balance precede and likely contribute to subsequent body weight divergence. Glucose tolerance tests revealed enhanced glucose clearance, and indices of insulin and leptin resistance were ameliorated in dKO mice (Fig. 1c–e). Computed tomography imaging demonstrated a significantly lower visceral adipose tissue (VAT; WT: 4417.9 ± 258.38 mm^3^; dKO: 3170.6 ± 300.83 mm^3^; *P* = 0.0345) to subcutaneous adipose tissue (SAT; WT: 1934.1 ± 134.70 mm^3^; dKO: 1754.8 ± 146.4 mm^3^; *P* = 0.465) (VAT/SAT; WT: 2.29 ± 0.096; dKO: 1.81 ± 0.107; *P* = 0.0251) ratio (n = 4; Fig. 1f, g). Histological analyses further confirmed decreased lipid accumulation in iWAT, eWAT, BAT, and liver (Fig. 1i, j, l, m, o, p, r, s).

### Reduced food intake and increased energy expenditure in NPGL/NPGM-dKO mice

To determine the behavioral and metabolic basis of the lean phenotype, we assessed food intake and energy expenditure. NPGL/NPGM-dKO mice consumed significantly less food than WT controls under both ND and HFD conditions (Fig. 1t; Supplementary Fig. 4b). Indirect calorimetry revealed elevated oxygen consumption (VO_2_, normalized to body weight) during the dark phase, whereas locomotor activity was unchanged (Fig. 1u, v), indicating that the increase in energy expenditure is not attributable to differences in physical activity. In addition, female NPGL/NPGM-dKO mice fed a high-fat diet exhibited reduced body weight and decreased food intake compared with WT females (Supplementary Fig. 5a, b). Whole-body oxygen consumption showed a modest increase, reaching statistical significance during the dark phase on the first day and the light phase on the second day of measurement (Supplementary Fig. 5c). These findings indicate that the anti-obesity phenotype is not restricted to male mice. Notably, increased energy expenditure in dKO mice is attributable to enhanced thermogenesis rather than increased physical activity.

### Altered hypothalamic neuropeptide and catecholaminergic signaling

**Fig. 2:**
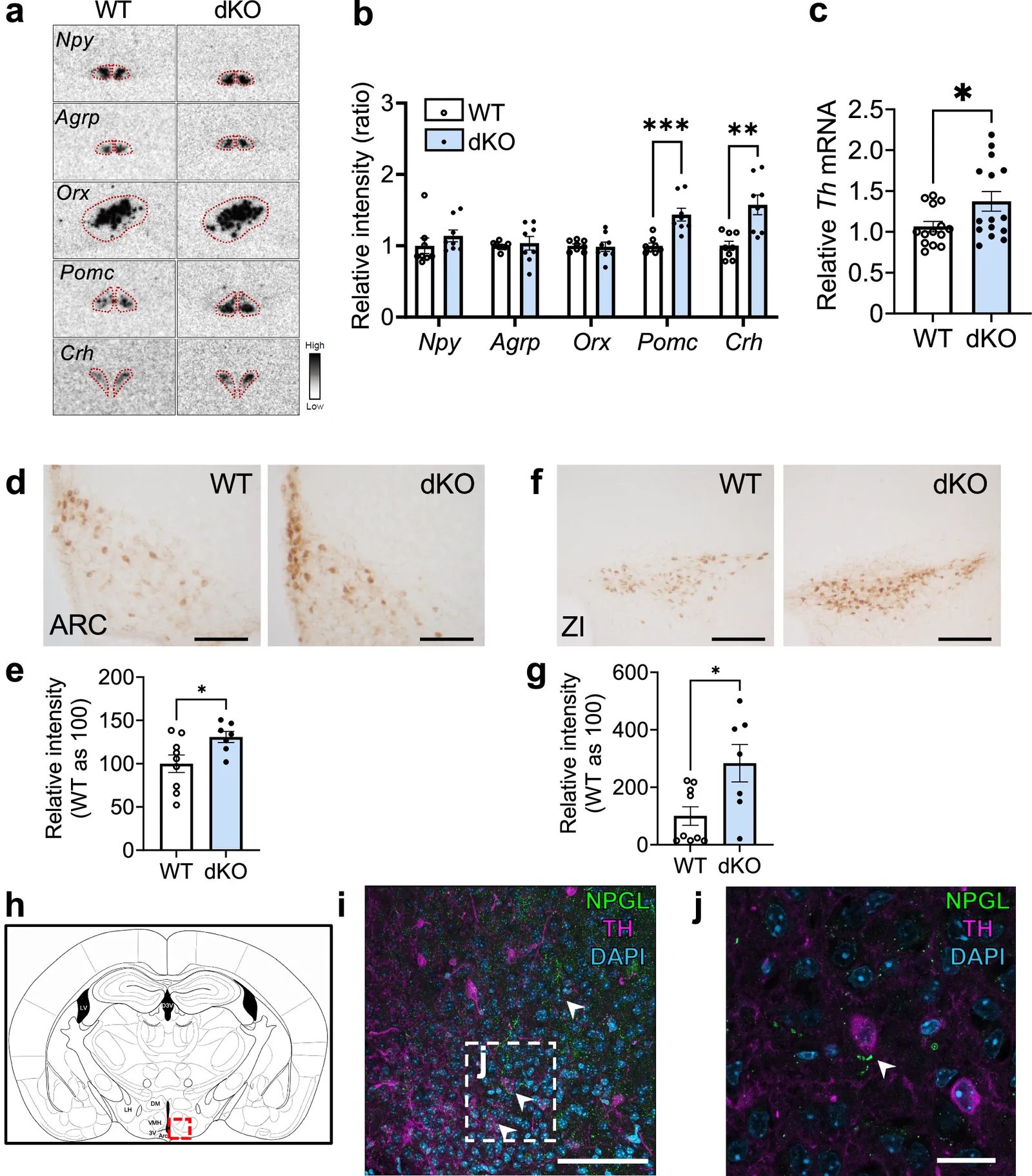
Relationship between the NPGL/NPGM system and neuropeptides related to feeding regulation. Wild-type (WT) or *Npgl*- and *Npgm*-double knockout (dKO) male mice were fed a normal diet (ND). **a** Representative images of in situ hybridization for genes encoding neuropeptides related to energy metabolism (*neuropeptide Y*, *Npy*; *agouti-related protein*, *Agrp*; *orexin*, *Orx*; *pro-opiomelanocortin*, *Pomc*; *corticotropin-releasing hormone*, *Crh*). **b** Relative intensity of mRNA expression of *Npy, Agrp*, *Orx*, *Pomc*, and *Crh* (*n* = 8). **c** Relative mRNA expression levels of *tyrosine hydroxylase* (*Th*) in the mediobasal hypothalamus measured by qPCR (*n* = 14 for WT and *n* = 15 for dKO mice). Representative immunohistochemistry images of TH in the arcuate nucleus of the hypothalamus (ARC) (**d**) and the zona incerta (ZI) (**f**). Scale bars: 50 μm (**d**), and 100 μm (**f**). Relative intensity of TH protein staining in the ARC (**e**) and the ZI (**g**) (*n* = 9 for WT and *n* = 7 for dKO mice). **h** Illustration of the mouse brain. The red square indicates the observed region in (**i**) and (**j**). Double immunostaining for NPGL (green), TH (magenta), and DAPI (blue) in the ARC. Scale bar: 100 μm (**i**) and 20 μm (**j**). The white square indicates the region of observation in (**j**). The arrowhead indicates TH-positive cells to which the NPGL neurons project. Data are presented as the mean ± SEM. For bar graphs, each dot represents data from an individual mouse. Asterisks refer to *P*-values obtained from an unpaired Student’s *t*-test (**b**, **c**, **e**, **g**) (*P < 0.05; **P < 0.01; ***P < 0.005). Exact *P*-values are indicated in the Source Data file.

Given the reduction in food intake, we examined hypothalamic expression of appetite-regulating neuropeptides. In situ hybridization revealed increased mRNA expression of *Pomc* and *Corticotropin-releasing hormone* (*Crh*), which encode anorexigenic factors, in the hypothalamus of NPGL/NPGM-dKO mice, whereas expression of orexigenic genes (*Npy*, *Agrp*, and *Orexin* (*Orx*)) remained unchanged (Fig. 2a, b; Supplementary Fig. 6a). Conversely, intracerebroventricular (ICV) administration of NPGL or NPGM suppressed *Pomc* expression in WT mice (Supplementary Fig. 6b, c), supporting a role for NPGL/NPGM neurons in inhibiting anorexigenic pathways. Because catecholaminergic circuits are critical regulators of feeding and energy expenditure^21–23^, we next assessed catecholamine-related markers, especially tyrosine hydroxylase (TH). *Th* mRNA expression was elevated in the mediobasal hypothalamus of dKO mice (Fig. 2c). Immunohistochemical analyses showed higher TH immunoreactivity in dKO mice of the arcuate nucleus, Zona Incerta (ZI), and locus coeruleus, but not in the ventral tegmental area (Fig. 2d–g; Supplementary Fig. 6d–g). Double-labeling analyses demonstrated projections from NPGL-expressing neurons to TH-positive cells in the arcuate nucleus (Fig. 2h–j), suggesting potential connectivity between NPGL/NPGM neurons and catecholaminergic pathways.

### Enhanced sympathetic activation and thermogenesis in brown adipose tissue

**Fig. 3:**
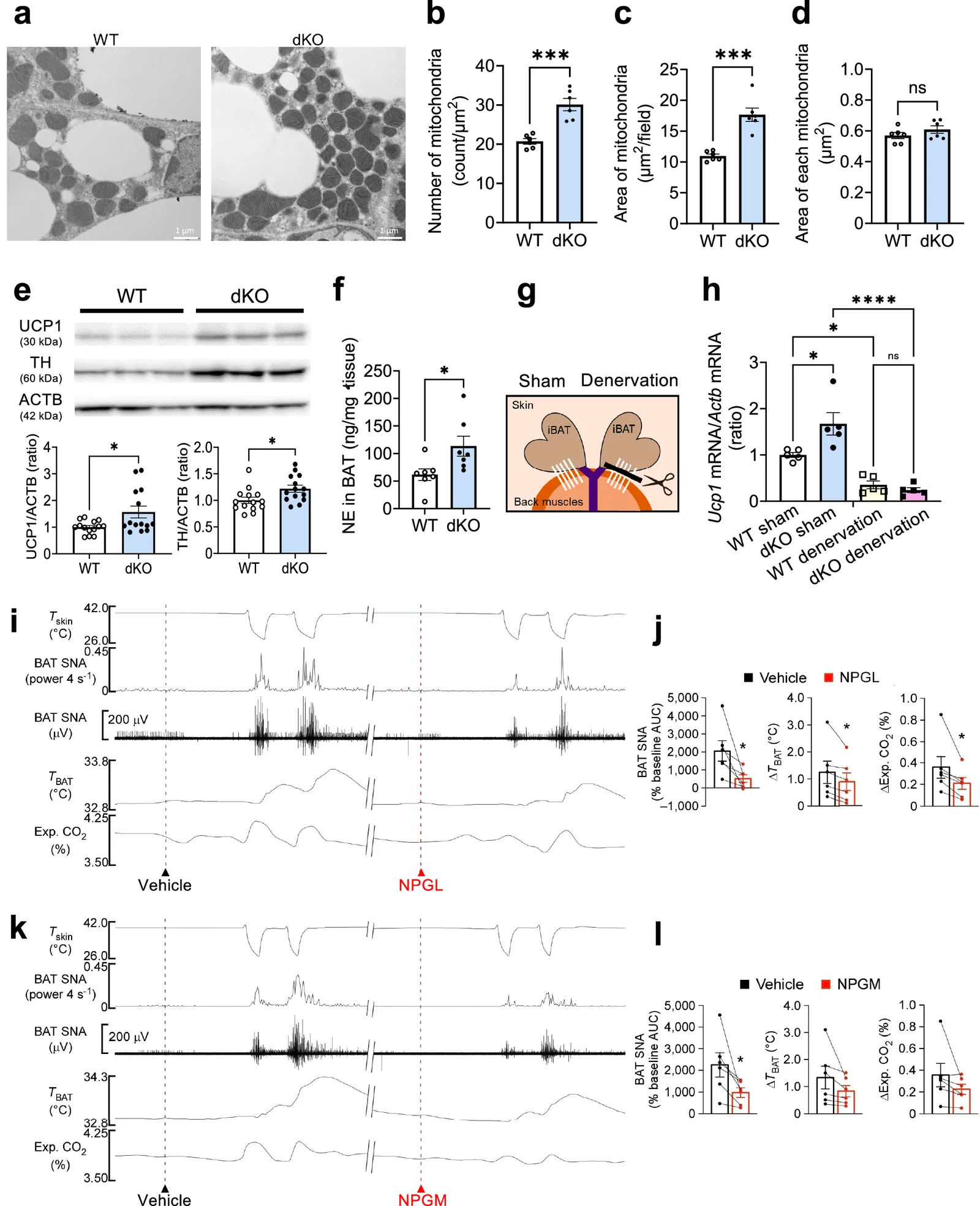
NPGL/NPGM deficiency enhances BAT thermogenesis and sympathetic activity. Wild-type (WT) or *Npgl-* and *Npgm*-double knockout (dKO) male mice were fed an ND. **a**–**d** Mitochondria in BAT were measured using a transmission electron microscope (*n* = 6). **a** Representative images of mitochondria in BAT of mice at 12 weeks of age. Scale bars: 1 μm. **b** Number of mitochondria in each field. **c** Total area of mitochondria in each field. **d** Average area of each mitochondrion. **e** Quantitative western blot analysis of uncoupling protein 1 (UCP1) and tyrosine hydroxylase (TH) (n = 14). Protein expression was normalized to that of β-actin (ACTB) (*n* = 14). **f** Norepinephrine (NE) contents in BAT (*n* = 7). **g** Schema of surgical denervation of interscapular BAT (iBAT) for unilateral denervation and contralateral sham operations in iBAT of 12-week-old mice. **h** Relative *Ucp1* expression levels after unilateral denervation in the BAT. mRNA expression was normalized to *Actb* mRNA levels (*n* = 5). **i**–**l** Effects of ICV injection of NPGL or NPGM on skin cooling-evoked BAT thermogenesis in WT rats. Changes in BAT sympathetic nerve activity (SNA), BAT temperature (*T*_BAT_) and expired CO_2_ (Exp CO_2_) in response to skin cooling (skin temperature, *T*_skin_) were measured following vehicle, NPGL **i**, **j** or NPGM injection (**k**, **l**) and compared statistically (*n* = 6). The waveform data shown correspond to the same experiments presented in Supplementary Fig. 8. Data are presented as the mean ± SEM. For bar graphs, each dot represents data from an individual animal. Asterisks indicate *P*-values determined by an unpaired Student’s *t*-test (**b**–**f**), paired t-test (**j**, **l**), or one-way ANOVA with Tukey’s multiple-comparisons test (**h**) (*P < 0.05; ***P < 0.005; ****P < 0.001; ns: not significant, *P* > 0.05). Exact *P*-values are provided in the Source Data file.

Because BAT thermogenesis is a major contributor to energy expenditure^24,25^, we examined BAT morphology and function. Histological analyses revealed smaller lipid droplets in BAT from NPGL/NPGM-dKO mice under both ND and HFD conditions (Fig. 1o, p; Supplementary Fig. 4j, k). Transmission electron microscopy showed increased mitochondrial density in BAT of dKO mice, without changes in mitochondrial size or morphology (Fig. 3a–d). Consistent with enhanced thermogenic capacity, expression of genes involved in mitochondrial function and thermogenesis, including *Cpt1a*, *Ucp1*, and *Prdm16*, was upregulated in BAT of dKO mice (Supplementary Fig. 7a). Mitochondrial gene expression (*Cytb1*, *Cox1*, and *Nd2*) was also increased (Supplementary Fig. 7b), and UCP1 protein abundance was elevated (Fig. 3e). To further assess the involvement of the hypothalamic–pituitary–thyroid axis in the thermogenic phenotype, we examined central and peripheral thyroid-related parameters. Hypothalamic *Trh* mRNA expression was not significantly different among wild-type, NPGL/NPGM double knockout, NPGL-injected, and NPGM-injected mice (Supplementary Fig. 6a–c). In addition, serum TSHβ levels and hypothalamic *Tsh* mRNA expression were comparable between wild-type and NPGL/NPGM dKO mice (Supplementary Fig. 7c–e). Consistent with these findings, circulating T4 levels were unchanged, indicating that thyroid axis activity is not altered by NPGL/NPGM deficiency or treatment, and suggesting that systemic thyroid hormone signaling is not a major contributor to the thermogenic phenotype observed in NPGL/NPGM-deficient mice.

Instead, indices of sympathetic nervous system (SNS) activity increased. TH protein levels in BAT and norepinephrine contents in BAT and iWAT were elevated in dKO mice (Fig. 3e, f; Supplementary Fig. 7f). Surgical denervation of BAT abolished the enhanced *Ucp1* expression in dKO mice (Fig. 3g, h), demonstrating that sympathetic input is required for the augmentation of their BAT thermogenesis. To further determine whether central NPGL and NPGM act upstream of the SNS, we assessed the effects of acute peptide administration. ICV administration of either NPGL or NPGM in WT rats diminished cold-evoked increases in BAT sympathetic nerve activity, BAT temperature, and expired CO₂, without affecting heart rate or mean arterial pressure (Fig. 3i–l; Supplementary Fig. 8). These results indicate that the central actions of NPGL/NPGM inhibit thermogenic sympathetic outflow.

### Acute suppression of NPGL/NPGM signaling and human genetic association

**Fig. 4:**
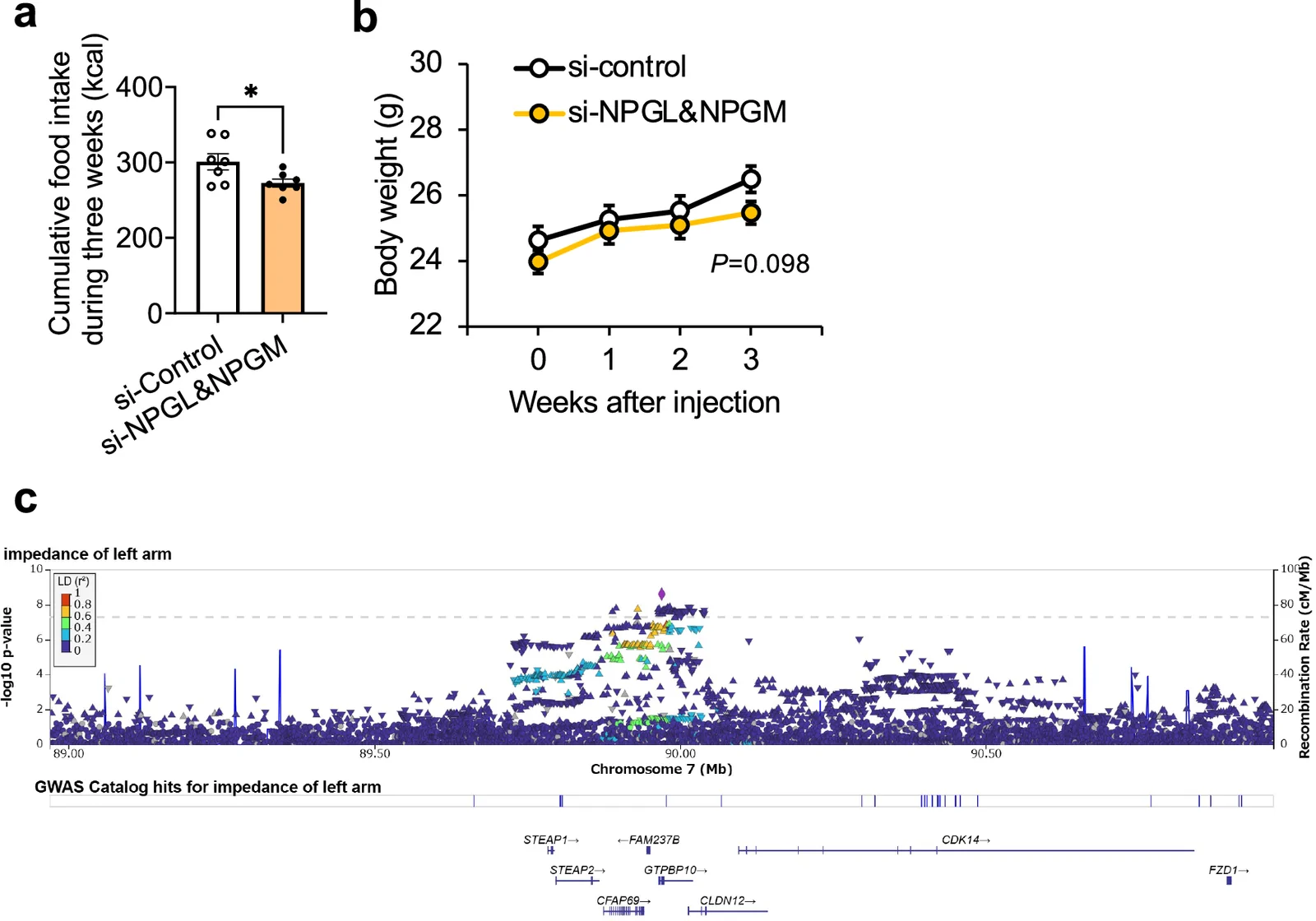
Therapeutic potential of NPGL/NPGM system in obese phenotype and human genetic analysis. **a**, **b** Silencing of *Npgl* and *Npgm* using small interfering RNA (siRNA) in wild-type (WT) mice fed a high-fat diet. **a** Cumulative food intake over 3 weeks (*n* = 7). **b** Body weight (*n* = 7). Data are presented as the mean ± SEM, and each dot represents data from an individual mouse. Asterisks refer to *P*-values obtained from an unpaired Student’s *t*-test (*P < 0.05). Exact *P*-values are indicated in the Source Data file. **c** Association between left arm impedance in humans and the *FAM237B* locus. A regional association plot for the impedance of the left arm near *FAM237B* is indicated by LocusZoom, based on summary statistics from the UK Biobank. The lead variant rs42637 is shown in purple, and the surrounding variants are color-coded according to their linkage disequilibrium (r^2^) with rs42367. Genomic coordinates were based on the GRCh37 reference genome.

To assess whether transient suppression of NPGL/NPGM signaling is sufficient to recapitulate aspects of the dKO phenotype, we performed siRNA-mediated knockdown in WT mice. This intervention reduced food intake and induced modest body weight loss; however, no significant changes in energy expenditure or locomotor activity were observed (Fig. 4a, b and Supplementary Fig. 9). These findings indicate that acute and partial suppression of *Npgl/Npgm* is not sufficient to fully reproduce the metabolic phenotype observed in constitutive dKO mice, suggesting that the magnitude and/or duration of gene suppression contributes to the phenotype.

**Fig. 5:**
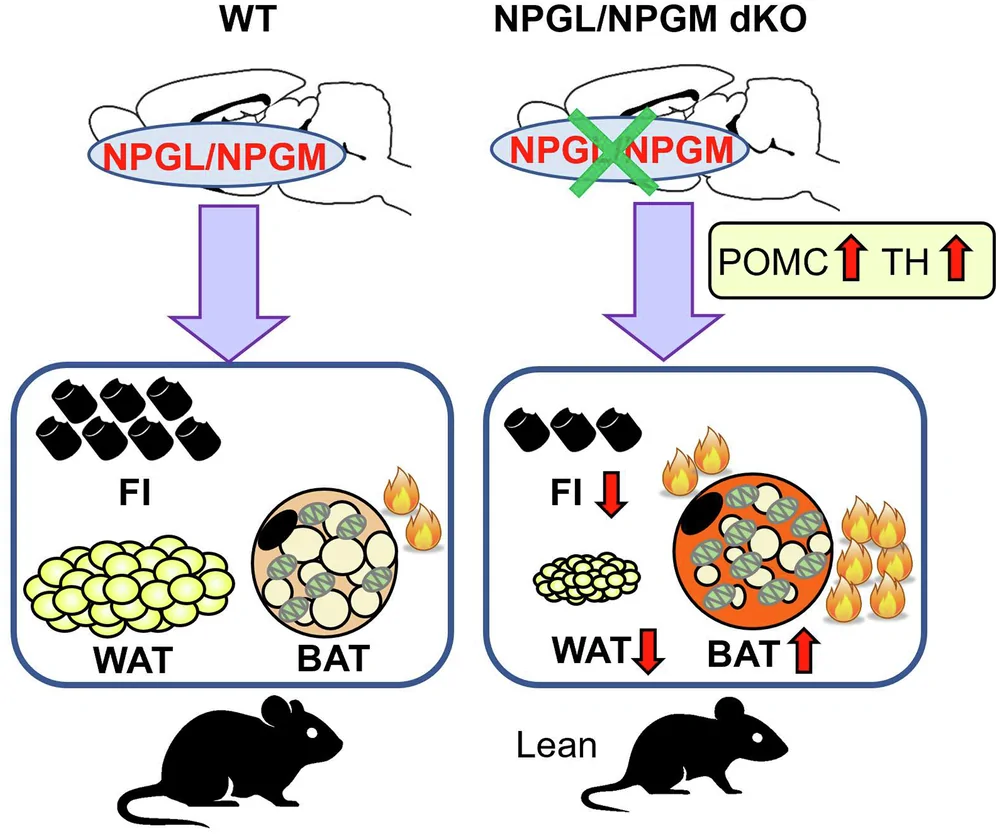
Proposed model illustrating how loss of NPGL/NPGM signaling influences energy balance. NPGL and NPGM are expressed in the arcuate nucleus (ARC) of the hypothalamus and exert tonic inhibitory effects on anorexigenic and catecholaminergic pathways. Loss of NPGL/NPGM signaling is associated with increased activity of pro-opiomelanocortin (POMC) neurons and enhanced catecholaminergic tone, leading to elevated sympathetic nervous system output. Increased sympathetic drive promotes activation of brown adipose tissue (BAT), characterized by increased uncoupling protein 1 (UCP1) expression and thermogenesis, thereby elevating energy expenditure. In parallel, enhanced hypothalamic anorexigenic signaling contributes to reduced food intake. Together, these coordinated central and peripheral changes are associated with a lean phenotype under both normal and high-fat diet conditions.

To explore potential relevance to human metabolic traits, we analyzed human genome-wide association data. A GWAS based on left arm impedance identified a lead variant (rs42637) located approximately 19 kb upstream of the *NPGM* (*FAM237B*) locus (Fig. 4c). Although no expression quantitative trait locus effect was detected, the proximity of this variant suggests possible cis-regulatory involvement. These observations provide preliminary genetic evidence of an association between the *NPGM* locus and human body composition traits. A schematic model summarizing the proposed mechanisms linking NPGL/NPGM signaling to appetite suppression and enhanced thermogenesis is shown in Fig. 5.

## Discussion

In this study, we provide comprehensive loss-of-function evidence of the role of the neurosecretory proteins NPGL and NPGM. We demonstrated that the deletion of NPGL and NPGM, which are mainly expressed in the ARC of the hypothalamus, results in a robust anti-obesity phenotype characterized by reduced food intake and enhanced energy expenditure. NPGL/NPGM-dKO mice were lean under both normal and HFD conditions and exhibited lower adiposity and improved glucose tolerance.

Mechanistically, these effects were associated with increased expression of anorexigenic neuropeptides in the hypothalamus, an elevated sympathetic tone, and enhanced UCP1-dependent thermogenesis in BAT, supporting a model in which hypothalamic anorexigenic pathways and BAT thermogenesis are jointly involved in the anti-obesity phenotype observed in NPGL/NPGM-dKO mice. Together, these data support the interpretation that alterations in energy balance precede body weight divergence, rather than representing secondary consequences of reduced adiposity. Together, these results substantially expand on those of previous gain-of-function studies. Previous studies have demonstrated that the central administration of NPGL or NPGM promotes food intake and lipogenesis, with NPGL known to modulate POMC neurons and increase fat accumulation^15,17–19^.

However, these studies relied exclusively on gain-of-function models based on exogenous peptide administration, which does not fully reflect the endogenous physiological roles. Previous studies have shown that *Npgl* overexpression in mice promotes adiposity, hyperinsulinemia, and hepatic steatosis, particularly with low-fat/medium-sucrose diets^26,27^. Similarly, Cre-dependent *Npgm* overexpression and the acute chemogenetic activation of NPGM neurons lead to increased food intake and transient body weight gains^16^. However, these effects are either diet-dependent or short-lived. Furthermore, the ablation of NPGM neurons impairs glucose tolerance, suggesting a complex and context-dependent role for these peptides^16^. In contrast, our dKO mice displayed consistent and long-lasting anti-obesity phenotypes, including reduced fat mass, improved glucose tolerance, and resistance to diet-induced obesity across dietary conditions. Notably, POMC and TH expression were upregulated in the hypothalamus of dKO mice, indicating that endogenous NPGL/NPGM continuously suppressed anorexigenic and catecholaminergic signaling. These results not only validate the metabolic relevance of NPGL and NPGM observed in the overexpression models but also highlight their essential physiological roles in maintaining energy storage and suppressing energy expenditure.

Mechanistically, NPGL/NPGM deficiency led to increased TH expression in the ARC, ZI, and LC nuclei, as well as increased NE and UCP1 expression in the BAT and inguinal WAT, indicating enhanced sympathetic tone. These effects were abolished after surgical BAT denervation, confirming the role of SNS activation in the lean phenotype. Furthermore, the acute ICV administration of NPGL or NPGM in rats diminished cold-evoked BAT thermogenesis without altering cardiovascular parameters, indicating the specific inhibition of thermogenic sympathetic outflow by these peptides. These findings suggest that changes in hypothalamic TH expression may be associated with the alteration of energy balance in NPGL/NPGM-deficient mice. Notably, although thyroid hormone signaling is a major regulator of energy expenditure, the absence of changes in hypothalamic *Trh* and *Tsh* expression, circulating TSHβ, and serum T4 levels argues against a major contribution of the thyroid axis to the metabolic phenotype observed in NPGL/NPGM-deficient mice.

Importantly, siRNA-mediated *Npgl* and *Npgm* knockdown in WT mice reproduced the hypophagic phenotype, demonstrating that acute suppression of these genes is sufficient to reduce feeding. In addition, siRNA-mediated knockdown resulted in only partial and acute reduction of *Npgl*/*Npgm* expression and did not fully reproduce the metabolic phenotype observed in dKO mice. This likely reflects differences in the magnitude and duration of gene suppression, and these results should be interpreted with caution. In parallel, an analysis of a large-scale GWAS dataset identified an obesity-associated SNP (rs42637) located approximately 19 kb upstream of the human *NPGM* (also known as *FAM237B*) locus. Although no direct expression Quantitative Trait Loci (eQTL) effect was detected, the proximity of this variant raises the possibility of a cis-regulatory effect on *NPGM* expression. To our knowledge, this represents the first report of a human obesity-associated variant located near the NPGM (FAM237B) locus. Although the functional significance of this association remains to be determined, it provides preliminary genetic evidence suggesting a potential link between the NPGL/NPGM system and human metabolic traits^15,16,20^.

Several limitations of this study should be acknowledged. Although we cannot completely exclude subtle developmental contributions, the absence of body weight differences in single knockout mice under chow conditions and the emergence of a lean phenotype primarily during HFD challenge argue against a primary developmental defect. Second, although NPGL and NPGM are predominantly expressed in the hypothalamus, transcriptomic studies have detected *Npgm* mRNA in additional brain regions and peripheral tissues, including the testes and BAT^15,28,29^. A recent Ribo-seq study identified *Npgm* as a small open-reading frame-encoded peptide in the BAT with orexigenic activity when administered centrally^19^. These findings highlight the possibility of tissue-specific functions, and future studies using region-specific KO strategies are required to clarify this. In addition, specific receptors for NPGL and NPGM have not yet been identified, although recent studies have implicated GPR83 as a candidate receptor^30–32^. *Gpr83*-KO mice show partial overlap with our NPGL/NPGM-dKO phenotype, including protection from HFD-induced obesity, but also exhibit increased ghrelin-responsiveness due to heterodimerization with the growth hormone secretagogue receptor^33^. Thus, the NPGL/NPGM–GPR83 axis may represent a signaling pathway with complex regulatory functions in appetite and metabolism. Further work using receptor- and region-specific genetic approaches will be required to clarify downstream signaling mechanisms. Furthermore, although our genome-wide association analysis identified an obesity-associated variant near the NPGM (FAM237B) locus, functional validation in human systems remains to be established. Accordingly, the translational implications should be interpreted with caution, although the proximity of this variant to NPGM suggests a potential link between NPGL/NPGM signaling and interindividual variation in body composition.

In summary, our findings define NPGL and NPGM as endogenous regulators of energy homeostasis that act through coordinated effects on feeding behavior and thermogenic energy expenditure. Loss of NPGL/NPGM signaling leads to a dual anti-obesity phenotype mediated by enhanced anorexigenic signaling and increased sympathetic activation of brown adipose tissue. These results provide a physiological framework for understanding NPGL/NPGM functions and suggest that further investigation of this system may inform future strategies targeting obesity and metabolic disorders.

## Methods

### Maintenance of mice and rats

C57BL/6 J and ICR mice were purchased from SLC (Shizuoka, Japan) and CLEA Japan (Tokyo, Japan). Mice were housed at 24 ± 2 °C under a 12:12 h light–dark cycle with ad libitum access to tap water and normal chow (CE-2; CLEA Japan). In several experiments, an HFD (High-fat diet 32; CLEA Japan) was also used. All animal procedures were performed in accordance with the Guide for the Care and Use of Laboratory Animals prepared by the Oita University. All animal experiments were performed in accordance with institutional and national guidelines, and the experimental protocol was approved by the review board committee of Oita University (approval numbers: 170302 and 230301). Slc: Wistar/ST rats were purchased from SLC and housed with ad libitum access to food and water at 25 ± 2 °C with a standard 12 h light/dark cycle. The Nagoya University Graduate School of Medicine was approved by the Nagoya University Animal Care and Use Committee (approval number: #M220097-002). We have complied with all relevant ethical regulations for animal use. All experiments were performed in male mice unless otherwise specified. The study was carried out in compliance with the ARRIVE guidelines.

### Establishment of NPGL- and NPGM-KO mice

*Npgl-* or *Npgm-KO* mice were generated using CRISPR–Cas9 technology. Two guide RNAs (gRNAs) were designed for each gene, targeting the first exon of *Npgl* (NC_000067.7) and the second exon of *Npgm* (NC_000071.7) using CRISPRdirect (https://crispr.dbcls.jp/) to delete coding sequences spanning the entire mature peptide region of NPGL and NPGM (Supplementary Fig. 1a). As a result, the mutant alleles lack the full mature peptide-coding sequence, which is essential for protein function. Although the genomic deletion affects part of the exon, Sanger sequencing confirmed disruption of the open reading frame, and the resulting alleles are predicted to produce truncated, non-functional proteins. Thus, these deletions effectively eliminate the functional mature peptide region and result in loss-of-function alleles. Mouse pronuclear-stage embryos were collected as previously described^34^. Female C57BL/6 J mice were superovulated via the intraperitoneal injection of 7.5 IU pregnant mare serum gonadotropin and 7.5 IU human chorionic gonadotropin 48 h later (Aska Pharmaceutical Co. Ltd., Tokyo, Japan), followed by mating with male C57BL/6 J mice. The following day, zygotes were harvested from the superovulated females and used for electroporation. Electroporation, to introduce the Cas9 protein and two gRNAs into intact mouse embryos, was performed using a previously reported technique for an animal KO system via electroporation. Briefly, 50–150 zygotes were electroporated in the presence of 40 μL of a Cas9/gRNA mixed solution using a NEPA 21 electroporator (NEPA GENE Co. Ltd., Chiba, Japan). The Cas9/gRNA solution was prepared by mixing custom crRNA for *Npgl* or *Npgm* gene targeting, generic tracrRNA (Integrated DNA Technologies [IDT], Coralville, IA, USA), and Alt-R S. p. HiFi Cas9 Nuclease V (Cas9) (IDT), as previously described. Embryos that reached the 2-cell stage were transferred to the oviducts of pseudopregnant female ICR mice (15–20 embryos per mouse). The edited genes in the offspring of the next generation were analyzed using Sanger sequencing to test for germline transmission. Sequencing confirmed disruption of the coding sequence, with no in-frame deletions detected among the established knockout lines, consistent with effective elimination of the mature peptide-coding region and functional loss of NPGL and NPGM. Heterozygous mice were intercrossed to generate homozygous *Npgl-* or *Npgm-*KO or dKO mice. For genotyping, genomic DNA was extracted from mouse tissues and amplified via PCR using the relevant primers. The primers and oligonucleotide sequences used for genome targeting are listed in Supplementary Table 1.

### Metabolic measurements and oral glucose tolerance tests

Sixteen-week-old male mice fed on an HFD for 8 weeks were fasted overnight, and blood was collected from decapitated mice at 10:00. The blood was centrifuged 4000 × *g*, 4 °C, for 15 min. The serum insulin, leptin, and TSH-beta concentration was determined with Morinaga Ultra Sensitive Mouse/Rat Insulin ELISA kit (Morinaga BioScience Inc., Yokohama, Japan), Mouse Leptin DuoSet ELISA (DY498, R&D Systems, Minneapolis, MN, USA), and LBIS TSH ELISA kit (296-88701, FUJIFILM Wako Pure Chemical Corp., Tokyo, Japan). For a glucose tolerance test, 16-week-old male mice fed on an HFD were fasted overnight, and glucose was administered intraperitoneally (1.0 mg/g body weight) using a previously described method^35^. The glucose levels were measured in blood withdrawn from the tail. Blood glucose was measured with a OneTouch VerioVue (LifeScan Japan, Tokyo, Japan).

### Microcomputed tomography (μCT) analysis of abdominal fat

Adipose tissue was quantified using the SkyScan 1276 X-ray μCT system (Bruker, Massachusetts, USA), as described previously^36^. Mice were scanned using the same parameters, as follows: a pixel size of 40 μm, a source voltage of 65 kV, a source current of 200 µA, 0.5 mm-thick aluminum filter, and a 0.4-degree rotation step. The total adipose tissue volume was determined between the lumbar vertebra 1 (L1) and the lumbar vertebra 6 (L6). Analyses of subcutaneous and intra-abdominal adipose tissues were based on the delimitation of the region of interest after 3D reconstruction of the scanned images. The 3D reconstructions and analysis of adipose tissue areas or volumes were performed using NRecon and CTAn software (Skyscan; Bruker).

### Metabolic chambers

O_2_ consumption and CO_2_ production were measured using an Oxymax-CLAMS system (Columbus Instruments, Ohio, United States) in accordance with the manufacturer’s instructions, as previously described^37^. Measurements of O_2_ and CO_2_ (mL/kg/h) were performed using an open-circuit indirect calorimetry system to calculate the difference between [O_2_] consumption and [CO_2_] production. Ambulatory activity was continuously assessed using infrared photocells along the X- and Z-axes. This was calculated when the animal broke through a series of infrared beams in sequence, thereby excluding stereotypy. All parameters were collected and analyzed using Oxymax-CLAMS software and continuously monitored throughout the 2 days of the experimental protocol. Energy expenditure (VO_2_) values were normalized to body weight using values automatically calculated by the Oxymax-CLAMS system.

### Western blotting

The detailed procedure for western blotting has been described elsewhere^38^. To detect TH and UCP1 levels in the interscapular BAT, western blot analysis, with each antibody, was conducted after SDS-PAGE. Tissues were homogenized in appropriate amounts of lysis buffer (100 mM KCl, 30 mM HEPES [pH 7.4], 5 mM MgCl_2_, 1% NP-40, 10% glycerol, 1 mM DTT, and 0.1 mM AEBSF). Proteins were separated using 10% SDS-PAGE and transferred onto Immobilon membranes (Merck Millipore, Burlington, MA, USA). Blots were blocked with 5% non-fat milk at room temperature for 30 min and subsequently incubated with primary antibodies targeting TH (1:1000; AB152, Sigma-Aldrich, St. Louis, MO, USA), UCP1 (1:1000; 14670S, Cell Signalling Technology, Massachusetts, USA), or β-actin (1:10,000; A3854, Sigma-Aldrich), overnight at 4 °C. After washing with TBST, the blots were incubated with appropriate horseradish peroxidase-conjugated secondary antibodies (1:10,000; AB2313567, Jackson ImmunoResearch, West Grove, PA, USA) at room temperature for 1 h. Detection was performed using a Chemi-Lumi One Super (Nacalai Tesque, Kyoto, Japan) and Fusion SOLO S (Vilber, Collegien, France). The antibodies used in this study are listed in Supplementary Table 4.

### Measurement of NE

The procedure for measuring NE has been described elsewhere^15,39^. BAT was removed from 16-week-old mice and stored at −80 °C until NE content measurements. To determine the NE contents in BAT, the tissue was homogenized in 0.2 M perchloric acid, placed on ice for 30 min, and centrifuged at 20,000 × *g* for 15 min at 0 °C. The supernatant was then subjected to liquid chromatography-tandem mass spectrometry (LC-MS/MS). The samples were processed using an LCMS-8040 spectrometer (Shimadzu Corporation, Kyoto, Japan) and analyzed based on standard curves.

### Real-time quantitative PCR (qPCR)

Total RNA extraction, cDNA synthesis, and qPCR were performed according to the manufacturer’s instructions, as previously described^15^. Total RNA was extracted using TRIzol reagent (Sigma-Aldrich). cDNA reverse transcription was performed using ReverTra Ace qPCR RT Master Mix (TOYOBO, Osaka, Japan). qPCR was conducted with THUNDERBIRD SYBR Master qPCR Mix (TOYOBO) on a LightCycler 96 system (Roche Diagnostics, Basel, Switzerland). The qPCR protocol was as follows: 95 °C for 3 min; followed by 45 cycles of 95 °C for 10 s, 60 °C for 30 s, and 72 °C for 1 s; and a final step of 95 °C for 10 s and 65 °C for 1 min. The generation of specific PCR products was confirmed through melting curve analysis. β-Actin was used as an internal control, and expression levels were compared using the ΔΔCt method. The primers used in this study are listed in Supplementary Table 2. Each data point represents an independent biological sample (individual animal), and no technical replicates were performed.

### Immunohistochemistry

The immunohistochemistry procedure has been described elsewhere^18^. Mice were anesthetized, and tissues were fixed with 4% paraformaldehyde (PFA) overnight after perfusion fixation and then incubated with 30% sucrose in phosphate-buffered saline until the samples sank to the bottom of the tube. Brain samples were then transversely embedded in Tissue-Tek OCT Compound (Sakura-Finetek, Tokyo, Japan) and fixed on dry ice. Serial sections were obtained using the Leica CM1950 microtome (Wetzlar, Germany). For immunohistochemical staining to detect TH, endogenous peroxidase activity was eliminated from the sections via incubation with 3% H_2_O_2_ in absolute methanol. After blocking nonspecific binding using 1% normal goat serum and 1% BSA in PBS containing 0.3% Triton X-100, the sections were incubated with an anti-TH rabbit antibody (1:1000; AB152, Sigma-Aldrich). The primary immunoreaction was performed through incubation with biotinylated goat anti-rabbit IgG (1:1000; VECTASTAIN ABC Rabbit IgG Kit, PK-4001; Vector Laboratories, Burlingame, CA, USA). The immunoreactive products were detected using an ABC kit (Vector Laboratories), followed by a diaminobenzidine (DAB) reaction using a Peroxidase Stain DAB Kit (25985-50, Nacalai Tesque). The DAB reaction was performed simultaneously under identical conditions (temperature, reaction time, and substrate concentration) across all tissues. The images were taken using a microscope (Axio Imager 2; Carl Zeiss, Germany). For the double immunohistochemical staining of NPGL/NPGM and NPGL/TH, permeabilization was performed using methanol. After blocking nonspecific binding using 1% normal donkey serum and 1% BSA in PBS containing 0.3% Triton X-100, the sections were incubated with the following primary antibodies: anti-NPGL rabbit antibody (1:250), anti-NPGM Guinea pig antibody (1:250) provided by Ukena^18,40^, and anti-TH mouse antibody (1:1000; MAB318, Merck Millipore). The polyclonal antibodies against NPGL and NPGM were generated using full-length synthetic peptides of the mature peptide region as immunogens, as previously described^18,40^. The sections were then incubated with the following secondary antibodies: Alexa Fluor 488 donkey anti-rabbit IgG (1:500; A21206, Invitrogen, Carlsbad, CA, USA), Alexa Fluor 594 donkey anti-mouse IgG (1:500; A21203, Invitrogen), and Alexa Fluor 594 donkey anti-guinea pig IgG (1:500; A11076, Invitrogen). DAPI fluoromount-G (0100-20; Southern Biotech, Birmingham, AL, USA) was used as the fluorescent mounting medium. The images were analyzed using a confocal microscope (LSM710 and LSM900; Carl Zeiss). Regions corresponding to the each nuclei were defined according to the mouse brain atlas^41^: the zona incerta (ZI) (approximately bregma −1.1 to −1.3 mm), the arcuate nucleus (ARC) (approximately bregma −1.6 to −1.8 mm), the lateral posterior arcuate nucleus (ArcLP) (approximately bregma −2.3 to −2.5 mm), the ventral tegmental area (VTA)/the substantia nigra pars compacta (SNc) (approximately bregma −2.8 to −3.0 mm), and the locus coeruleus (LC) (approximately bregma −5.3 to −5.5 mm). One image was captured and analyzed for each region. For quantitative analysis of TH immunoreactivity, signal intensity within defined regions of interest (ROI) was measured using ImageJ software (National Institutes of Health, Baltimore, MD, USA). Sections from all experimental groups were processed in parallel, and images were acquired under identical microscope settings, and values were normalized to the mean of the WT group. Because immunohistochemical staining intensity can be influenced by experimental conditions, these measurements should be interpreted as semi-quantitative rather than absolute estimates of protein abundance. The antibodies used in this study are listed in Supplementary Table 4.

### In situ hybridization (ISH)

The procedure for the ISH is described elsewhere^42^. Briefly, brain samples were collected after decapitation and then frozen on dry ice. The brain was cut with a microtome into 12 μm sections. Radioactively labeled deoxyoligonucleotide complementary probe sequences to transcripts encoding *Npy*, *Agrp*, *Orx*, *Pomc*, and *Crh* were used. After hybridization, hybridized sections were exposed for autoradiography (Hyperfilm, Amersham, Bucks, UK). Autoradiographic signals were quantified semiquantitatively using ImageJ (National Institutes of Health) software from anatomically matched regions of interest. Regions corresponding to the hypothalamic nuclei were defined according to the mouse brain atlas^41^: the PVH (approximately bregma −1.1 to −1.3 mm) and the ARC (approximately bregma −1.6 to −1.8 mm). One image was captured and analyzed for each region. Signal intensity within each ROI was measured and values were normalized to WT controls.

### Hematoxylin and eosin staining

White and brown adipose tissues and livers were soaked in 4% PFA at the end of the experiments and embedded in paraffin. Serial 10 µm sections were obtained using a Sliding microtome (SM2000R, Leica). Sections were air-dried and delipidated with xylene. The nuclei and cytoplasm were stained with hematoxylin and eosin (5 min for each stain), and the sections were washed with tap water. After dehydration with an alcohol series and clearance with xylene, the sections were mounted on slides and examined under a microscope (Axio Imager 2; Carl Zeiss). One image was captured and analyzed from one mouse. The size of adipocytes and lipid droplets in the obtained image was analyzed using Image-J software (National Institutes of Health).

### Electron microscopy

Elemental analysis of the mitochondria of BAT was performed using a transmission electron microscope (TEM, H-7650, HITACHI, Tokyo, Japan) equipped with an energy-dispersive X-ray microanalyzer (EDAX, Mahwah, NJ, USA) as previously described^43^. To prepare specimens for elemental analysis, tissues were fixed in 4% PFA and 2% glutaraldehyde and then in 1% osmium tetroxide and coated with gold, palladium, and osmium. Mitochondrial number was quantified within entire TEM microscopic fields of view acquired under identical magnification and imaging conditions. For each sample, five non-overlapping fields from independent sections were randomly selected, and all mitochondria visible within each field were counted. The size and number of mitochondria in the obtained image were analyzed using Image-J software (National Institutes of Health).

### Acute *ICV* injection in mice

ICV injection of NPGL or NPGM into the lateral ventricles of C57BL/6J mice was performed according to our established procedure^18^. For the acute injection of NPGL or NPGM (1 nmol each), a guide cannula (24 gauge, C316GS; Plastics One, Roanoke, VA, USA) was implanted unilaterally into the left lateral ventricle. The cannula was fixed to the skull using an acrylic resin. The final coordinates of the cannula tips were 0.5 mm caudal to the bregma, 0.1 mm lateral to the midline, and 1.25 mm ventral to the skull surface. The injector (31 gauge, C316IS; Plastics One) was extended 1.0 mm below the tips of the guide cannula. The injections were delivered using a Hamilton syringe connected to polyethylene tubing. The reagents were injected after a postoperative period of 1 week. The mice were decapitated 90 minutes after ICV injection, and the mediobasal hypothalamus was rapidly collected and snap-frozen in liquid nitrogen for qPCR analysis. To knockdown *Npgl* and *Npgm* in the hypothalamus, we performed in vivo siRNA transfection using C57BL/6 J mice, as previously described^44^. siRNA was administered into the third cerebral ventricle using a Hamilton syringe and a stereotaxic apparatus (coordinates: AP, 1.0 mm posterior to the bregma; L, 0 mm; V, 5.0 mm). For each gene (*Npgl* and *Npgm*), two independent siRNA sequences (Merck Millipore) were initially tested, and the sequence with the highest knockdown efficiency was selected for subsequent experiments. The siRNA sequences used in this study are listed in Supplementary Table 3. Antisense constructs contained three LNAs at the 3’ end of the siRNA. Knockdown efficiency was assessed by qPCR analysis of mediobasal hypothalamic tissue, showing approximately 40% and 60% reductions in *Npgl* and *Npgm* mRNA levels, respectively. Because of the limited sample size (*n* = 3), these analyses were used to confirm knockdown efficiency but were not subjected to statistical evaluation. According to the manufacturer’s instructions, siRNA (10 pg/μL) was mixed with Complexation Buffer and Invivofectamine 3.0 Reagent (Invitrogen, IVF3001), incubated at 50 °C for 30 min, and diluted with PBS. A total volume of 2 μL was injected into the third ventricle. After injection, mice were returned to their cages with ad libitum access to food and water.

### In vivo physiological recordings and ICV injection in anesthetized rats

In vivo physiological recordings using rats were performed as previously described^45^. Under gas anesthesia with 2% isoflurane in 100% O_2_, male rats (Slc:Wistar/ST, 300–400 g; SLC Japan) underwent cannulation of the femoral artery, femoral vein, and trachea, and then were transitioned to i.v. urethane (0.8 g kg^–1^) and α-chloralose (80 mg kg^–1^) anesthesia. Arterial pressure and heart rate were recorded using a pressure transducer attached to the arterial cannula. Body core temperature (*T*_core_) was monitored using a copper constantan thermocouple inserted into the rectum. To monitor skin temperature, the trunk was shaved, and a thermocouple was taped onto the abdominal skin. The trunk was then wrapped with a plastic water jacket, which was perfused with warm or cold water to maintain *T*_core_ at 36.0–38.0 °C. The rats were positioned in a stereotaxic apparatus with a spinal clamp, paralyzed with D-tubocurarine (initial dose: 0.6 mg IV, supplemented at 0.3 mg h^−1^), and artificially ventilated with 100% O_2_ (60 cycles min^−1^, tidal volume: 3.5 mL) to stabilize BAT nerve recording by preventing respiration-related movements and cooling-induced shivering. Mixed expired CO_2_ was measured using a capnometer (OLG-2800, Nihon Kohden, Tokyo, Japan), to provide an index of changes in whole-body metabolism, and was maintained at 3.5–4.5% under basal conditions. A needle-type thermocouple (0.33 mm diameter; Physitemp, Clifton, NJ, USA) was inserted into the left interscapular BAT pad to monitor the BAT temperature. Postganglionic BAT SNA was recorded from the central cut end of a nerve bundle isolated from the ventral surface of the right interscapular BAT pad after dividing it along the midline and reflecting it laterally. The nerve bundle was placed on bipolar hook electrodes under mineral oil. BAT SNA was filtered (1–300 Hz) and amplified (×5000–50,000) using a CyberAmp 380 (Axon Instruments, Union City, CA, USA). All physiological variables were digitized and recorded on a computer hard disk using Spike 2 software (CED, Cambridge, UK). A small hole was made on the dorsal skull (0.8 mm caudal to bregma and 1.8 mm lateral to the midline), and a 10 μL microsyringe, for ICV injection, was perpendicularly inserted into the hole to a depth of 3.0–4.0 mm from the brain surface to access the lateral ventricle. When the physiological parameters were stable, and BAT SNA was quiescent, 5 μL of vehicle, NPGL, or NPGM (5 nmol each) was injected over 1 min. Approximately 5 min after the completion of the injection, the trunk skin was cooled by 10 °C from approximately 40 °C, via perfusion of the water jacket with ice-cold water, and then rewarmed by switching to perfusion with warm water. A skin-cooling episode of the same intensity was sequentially administered twice. Physiological responses to skin cooling were recorded and quantitatively analyzed.

### Genetic analysis of the human *FAM237B* locus

To investigate the potential link between the *FAM237B* (also known as *NPGM*) locus and lipid metabolism traits, we used the Open Targets Genetics platform^46^ to explore genetic variants proximal to *FAM237B*. The GWAS summary statistics for relevant traits were retrieved, and the genomic region surrounding *FAM237B*, including the lead variant rs42637 identified in a GWAS of left-arm impedance, was visualized using LocusZoom^47^. Genomic coordinates and variant–gene distances were calculated using the GRCh37 reference genome.

### Statistical analysis and Reproducibility

Comparisons between WT mice and experimental mice were made using unpaired Student’s *t*-tests, paired *t*-test, or two-way analysis of variance (ANOVA) followed by Bonferroni’s multiple comparison test, as appropriate. Data from four groups were analyzed using one-way ANOVA with Tukey’s post-hoc test. Statistical significance was set at *P* < 0.05. Exact *P*-values are provided in the Source Data file. All experiments were performed using independent biological replicates (individual animals), and each data point represents a single animal. Information on sample size can be found in the figure legends. For qPCR analyses, each biological sample was analyzed in technical triplicate, and the mean value was used for statistical analysis. Analyses were performed using GraphPad Prism software (version 9 and 10; GraphPad Software, Inc., San Diego, CA, USA). All results are presented as the mean ± standard error of the mean (SEM). No data points were excluded from analysis unless a predefined technical failure occurred (e.g., assay failure or sample processing error). All collected data were included in statistical analyses.

### Reporting summary

Further information on research design is available in the Nature Portfolio Reporting Summary linked to this article.

## Supplementary information

## Data Availability

All data needed to evaluate the findings of this study can be found within the article, extended data or supplementary tables. Source data are provided within Supplementary Data 1.

## Code availability

This paper does not report original code.
