# Supplementary material for: Deletion of neurosecretory proteins GL and GM drives dual anti-obesity effects via appetite suppression and enhanced energy expenditure
Source: Commun Biol. 2026 Jun 3;9:1168. doi: 10.1038/s42003-026-10405-7 (PMC13538515; doi:10.1038/s42003-026-10405-7)
Supplement: Supplementary file 1 [file 42003_2026_10405_MOESM1_ESM.pdf]

Title

Deletion of neurosecretory proteins GL and GM drives dual anti-obesity effects via appetite suppression and enhanced energy expenditure

Authors

Kenshiro Shikano [1], Mitsuhiro Yoshimura [2], Takaki Yahiro [3, 4], Taishi Kurakazu [5], Shigeyoshi Saito [6], Hitoshi Teranishi [1], Ryoko Higa [1, 7], Ryohei Umeda [1, 8], Magdeline E. Carrasco Apolinario [1], Takashi Maruyama [2], Atsuo T. Sasaki [9, 10, 11], Toshikatsu Hanada [12], Takatoshi Hikida [13], Kazuyoshi Ukena [14], Josef M. Penninger [15, 16, 17, 18], Yoichi Ueta [2], Chikashi Terao [5, 19, 20, 21], Kazuhiro Nakamura [3] and Reiko Hanada [1*]

Affiliations

[1] Department of Physiology, Faculty of Medicine, Oita University, Yufu, Oita 879-5593, Japan

[2] Department of Physiology, School of Medicine, University of Occupational and Environmental Health, Kitakyushu, Japan.

[3] Department of Integrative Physiology, Nagoya University Graduate School of Medicine, Nagoya, 466-8550, Japan.

[4] Vollum Institute, Oregon Health and Science University, Portland, OR, 97239, U.S.A.

[5] Laboratory for Statistical and Translational Genetics, RIKEN Center for Integrative Medical Sciences, 1-7-22 Suehiro-cho Tsurumi-ku, Yokohama, Kanagawa, Japan.

[6] Department of Medical Physics and Engineering, Division of Health Sciences, The University of Osaka Graduate School of Medicine, Suita, Osaka, 560-0871, Japan.

[7] Department of Medicine, New York University Grossman School of Medicine, New York, NY, USA.

[8] Department of Advanced Medical Science, Faculty of Medicine, Oita University, Oita, Japan

[9] Department of Internal Medicine, University of Cincinnati College of Medicine, Cincinnati, 45242, USA

[10] Institute for Advanced Biosciences, Keio University, Tsuruoka, 997-0017, Japan

[11] Department of Clinical and Molecular Genetics, Hiroshima University, Hiroshima, 734-8551, Japan

[12] Department of Biochemistry and Molecular Genetics, Faculty of Medicine, Oita University
Yufu, Oita 879-5593, Japan

[13] Laboratory for Advanced Brain Functions, Institute for Protein Research, Osaka University, Osaka, Japan

[14] Laboratory of Neurometabolism, Graduate School of Integrated Sciences for Life, Hiroshima University, Higashi-Hiroshima, Hiroshima 739-8521, Japan

[15] Institute of Molecular Biotechnology of the Austrian Academy of Sciences, Vienna, Austria.

[16] Department of Medical Genetics, Life Sciences Institute, University of British Columbia, Vancouver, British Columbia, Canada.

[17] Helmholtz Centre for Infection Research, Braunschweig, Germany.

[18] Department of Laboratory Medicine, Medical University of Vienna, Vienna, Austria.

[19] Clinical Research Center, Shizuoka General Hospital, 4 Chome-27-1 Kitaando Aoi Ward, Shizuoka, Japan.
[20] The Department of Applied Genetics, The School of Pharmaceutical Sciences, University of Shizuoka, 52-1 Yada Suruga Ward, Shizuoka, Japan.
[21] Department of Statistical Genetics, Center for Genomic Medicine, Research Promotion Headquarters, Fujita Health University, 1-98 Dengakugakubo, Kutsukake-cho, Toyoake, Aichi 470-1192, Japan.

[*]**Correspondence and requests for materials should be addressed to**
Reiko Hanada M.D., Ph.D.
Department of Neurophysiology, Faculty of Medicine, Oita University,
Idaigaoka, Yufu City, Oita 879-5593, Japan
Tel: +81 97 586 5642; Fax: +81 97 586 5649
E-mail: reiko-hanada@oita-u.ac.jp

SUPPELEMENTARY FIGURES, TABLES, AND ANTIBODY'S LIST

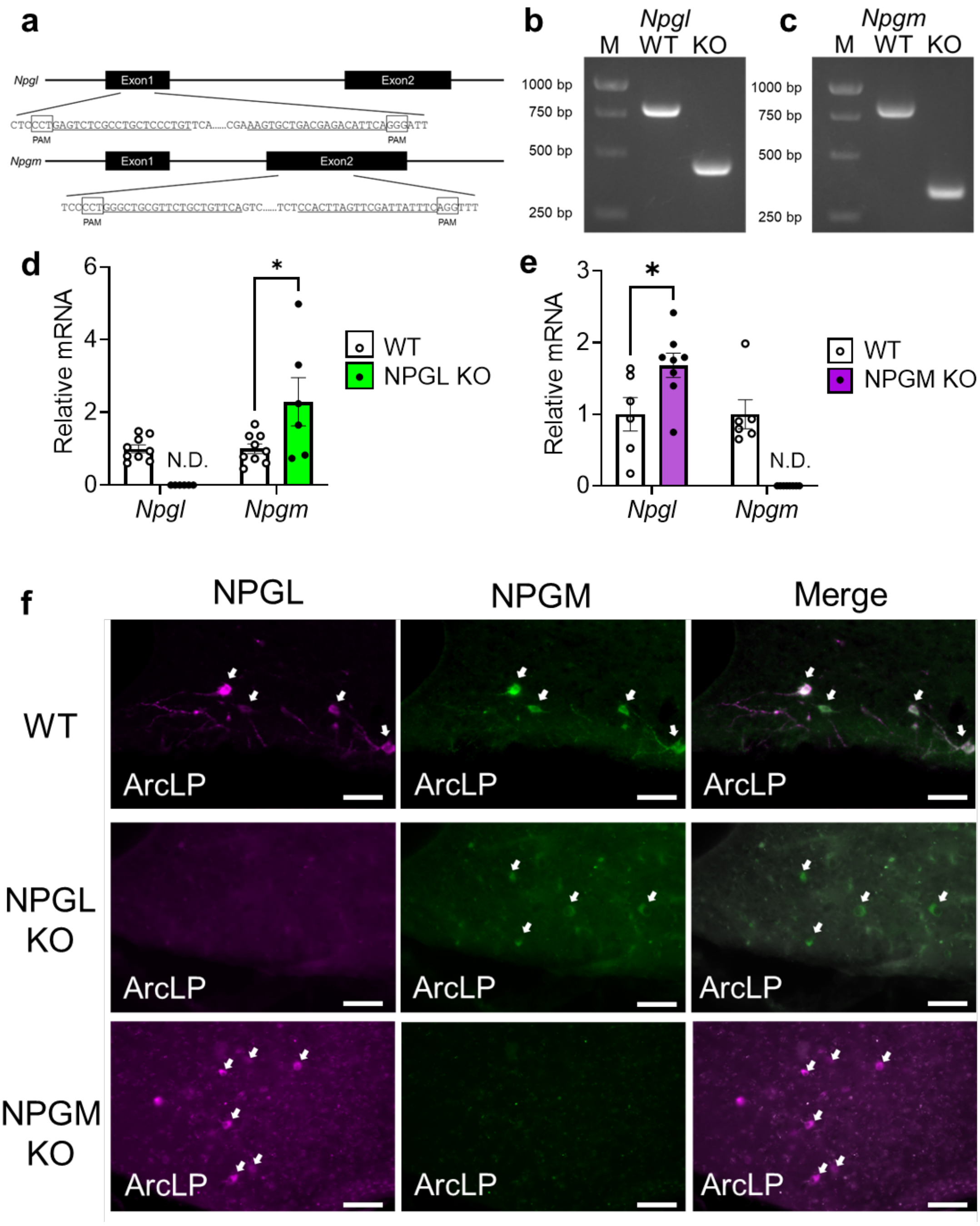

Supplementary Figure 1. Generation of NPGL- and NPGM-knockout mice.

Generation of *Npgl*- or *Npgm*-knockout (KO) mice using CRISPR/Cas9 technology. **a**, Design of two guide RNAs (gRNAs) for the *Npgl* or *Npgm* gene. The sequences of the gRNA are underlined. The square shows the sequences of the protospacer-adjacent motif (PAM). **b**, **c**, Small PCR products showing the deletion of *Npgl* (**b**) or *Npgm* (**c**). **d,** Quantitative PCR analysis of *Npgl* mRNA expression in the mediobasal hypothalamus of wild-type (WT) and *Npgl*-single KO mice (n = 9 for WT; n = 6 for KO). **e**, Quantitative PCR analysis of *Npgm* mRNA expression in the mediobasal hypothalamus of WT and *Npgm*-single KO mice (n = 6 for WT; n = 8 for KO).

In both **d** and **e**, transcript levels were significantly reduced or not detected in knockout animals, consistent with disruption of normal gene expression. N.D., not detected. Each data point represents an independent biological replicate (individual mouse). For qPCR analyses, RNA was extracted from each animal individually and analyzed separately. **f**, Representative images of immunohistochemistry against the NPGL (magenta) or NPGM (green) protein in the lateroposterior arcuate nucleus (ArcLP) of WT, *Npgl*-single KO, and *Npgm*-single KO mice. The arrows indicate NPGL- or NPGM-immunoreactive cells. Scale bars: 50 μm.

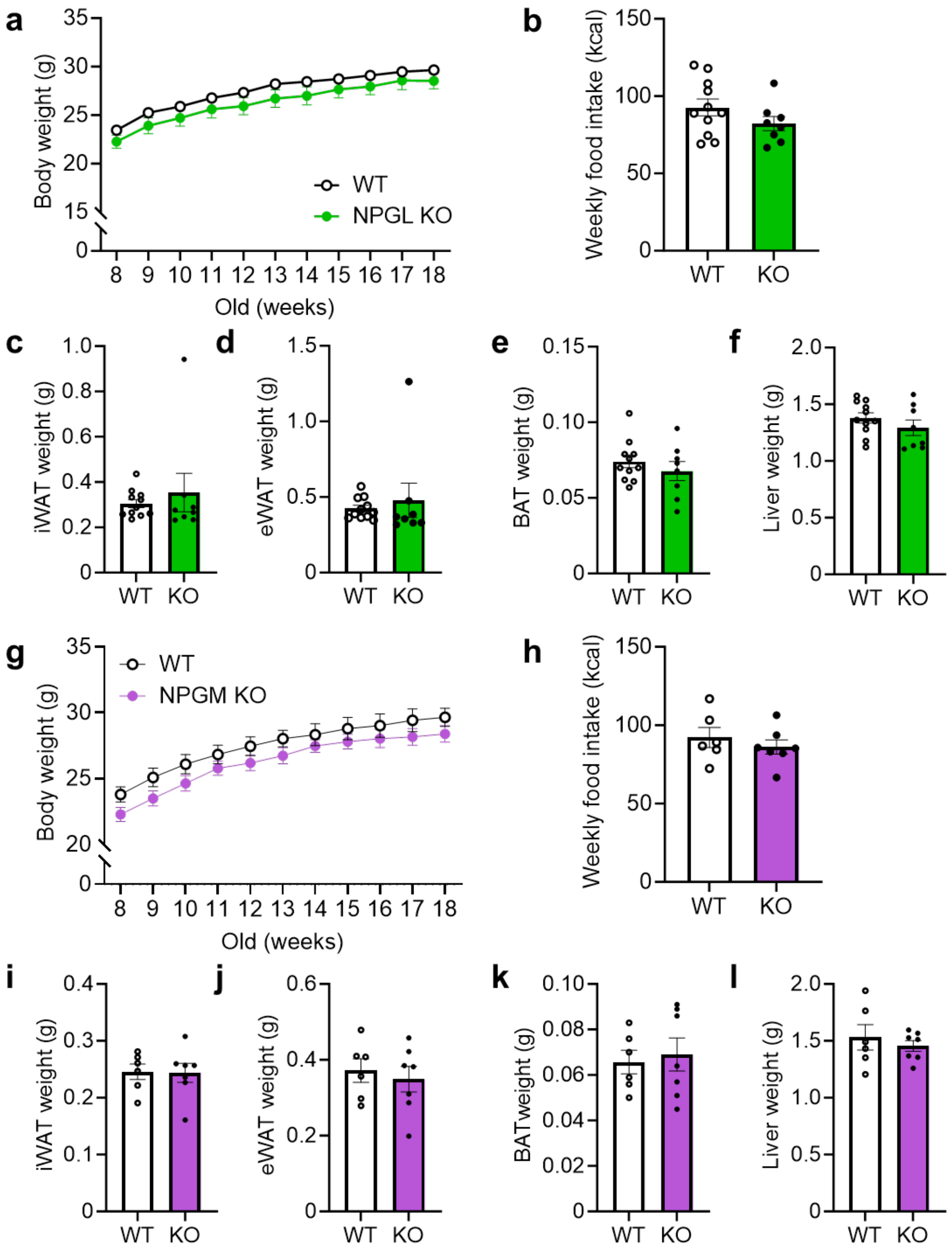

Supplementary Figure 2. Phenotype of *Npgl*- and *Npgm*-single knockout mice under normal diet (ND) conditions.

Wild-type (WT) and *Npgl*- and *Npgm*-single knockout (KO) male mice were fed an ND. **a**, Body weight. **b**, Weekly food intake at 12 weeks of age. **c**–**f**, Tissue weights of inguinal white adipose tissue (iWAT) (**c**), epididymal white adipose tissue (eWAT) (**d**), brown adipose tissue (BAT) (**e**), and the liver (**f**) (n = 11 for WT and n = 8 for NPGL KO mice). **g**, Body weight. **h**, Weekly food

intake at 12 weeks of age. **i–l**, Tissue weights iWAT (**i**), eWAT (**j**), BAT (**k**), and the liver (**l**) (n = 6 for WT and n = 7 for NPGM KO mice). Data are presented as the mean ± SEM. For bar graphs, each dot represents data from an individual mouse. Asterisks refer to *P*-values obtained via two-way ANOVA adjusted based on Bonferroni's multiple comparison test (**a**, **g**) or an unpaired Student's *t*-test (**b–f**, **h–l**). Exact *P*-values are indicated in the Source Data file.

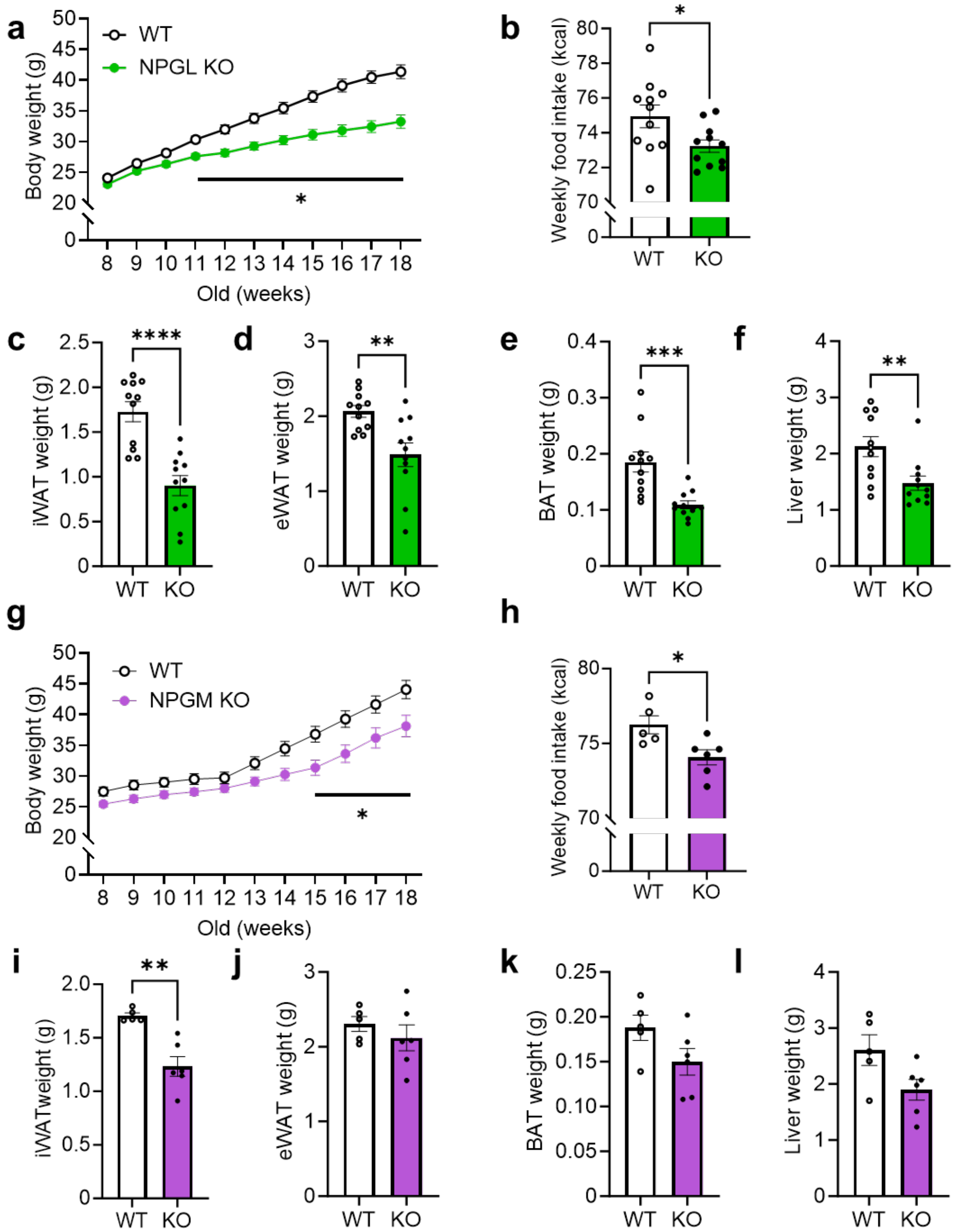

Supplementary Figure 3. Phenotype of NPGL- or NPGM-single knockout (KO) mice under high-fat diet (HFD) conditions.

a–**f**, Wild-type (WT) or *Npgl*-single KO male mice fed an HFD from 8 weeks of age (n =11). **a**, Body weight. **b**, Weekly food intake at 12 weeks of age. **c**–**f**, Tissue weights of inguinal white adipose tissue (iWAT) (**c**), epididymal white adipose tissue (eWAT) (**d**), brown adipose tissue (BAT) (**e**), and liver (**f**). **g**–**l**, WT or *Npgm*-single KO male mice were fed an HFD from 8 weeks of age (n = 5 for WT and n = 6 for NPGM-KO mice). **g**, Body weight. **h**, Weekly food intake at

12 weeks of age. **i–l**, Tissue weights of iWAT (**i**), eWAT (**j**), BAT (**k**), and liver (**l**). Data are presented as the mean ± SEM. For bar graphs, each dot represents data from an individual mouse. Asterisks refer to *P*-values obtained via two-way ANOVA adjusted based on Bonferroni's multiple comparison test (**a**, **g**) or an unpaired Student's *t*-test (**b–f**, **h–l**) (**P* < 0.05; ***P* < 0.01; ****P* < 0.005; *****P* < 0.001). Exact *P*-values are indicated in the Source Data file.

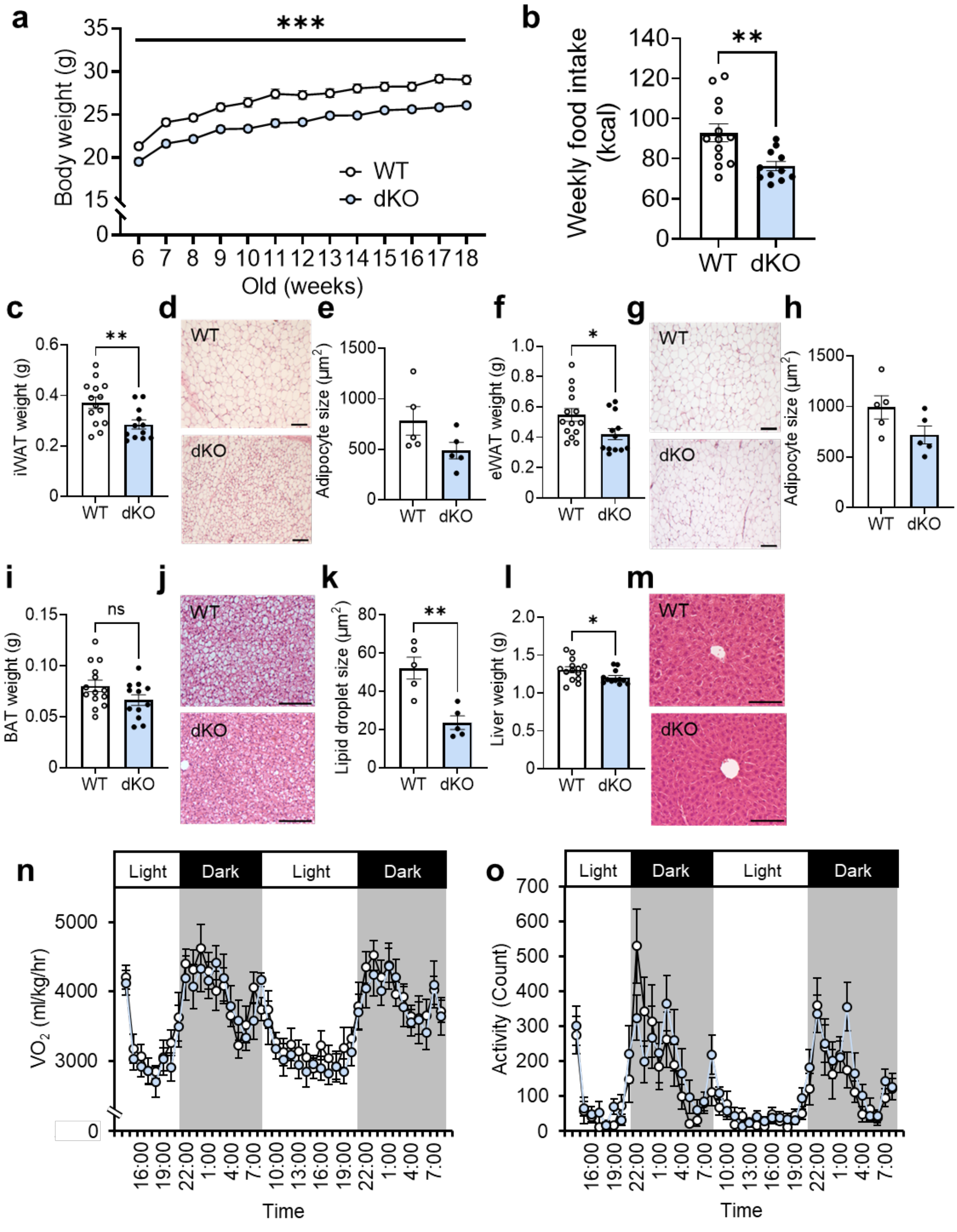

Supplementary Figure 4. Phenotype of *Npgl*- and *Npgm*-double knockout (dKO) mice under normal diet (ND) conditions.

Wild-type (WT) or *Npgl*- and *Npgm*-double knockout (dKO) male mice were fed an ND. **a**, **b**, Body weight (**a**) (n = 14 for WT and n = 12 for dKO mice) and weekly food intake from 12 to 14 weeks of age (**b**) (n = 13 for WT, and n = 11 for dKO mice). **c**–**m**, Tissue weights (n = 14 for WT and n = 12 for dKO mice), representative images of HE staining, and adipocyte or lipid droplet size (n = 5) of inguinal white adipose tissue (iWAT) (**c**–**e**), epididymal white adipose tissue

(eWAT) (**f**–**h**), brown adipose tissue (BAT) (**i**–**k**), and the liver (**l**, **m**). Scale bars: 100 μm. **n**, **o**, Whole-body oxygen consumption (VO_2) (**n**) and locomotor activity (**o**) in 16-week-old mice (n = 10). Data are presented as the mean ± SEM. For bar graphs, each dot represents data from an individual mouse. Asterisks refer to *P*-values obtained via two-way ANOVA adjusted based on Bonferroni's multiple comparison test (**a**, **n**, **o**) or an unpaired Student's *t*-test (**b**, **c**, **e**, **f**, **h**, **i**, **k**, **l**) (*$P < 0.05$; **$P < 0.01$; ***$P < 0.005$; ns: not significant, $P > 0.05$). Exact *P*-values are indicated in the Source Data file.

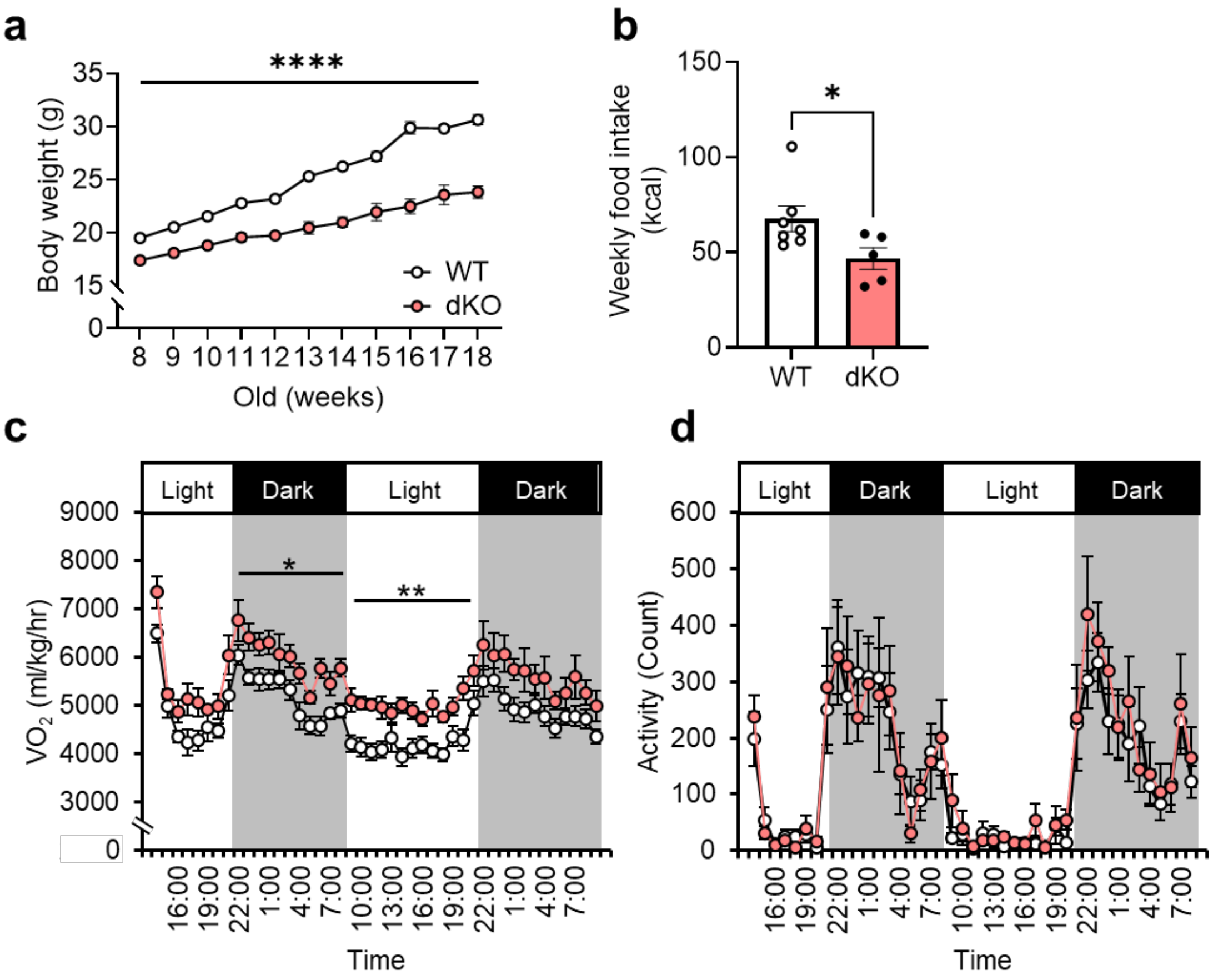

Supplementary Figure 5. Phenotype of female *Npgl*- and *Npgm*-double knockout (dKO) mice under high-fat diet (HFD) conditions.

Wild-type (WT) or *Npgl*- and *Npgm*-double knockout (dKO) female mice were fed an HFD from 8 weeks of age (n = 7 for WT and n = 5 for dKO mice). **a**, **b**, Body weight (**a**) and weekly food intake from 16 weeks of age (**b**). **c**, **d**, Whole-body oxygen consumption (VO_2) (**c**) and locomotor activity (**d**) in 16-week-old mice. Data are presented as the mean ± SEM. For bar graphs, each dot represents data from an individual mouse. Asterisks refer to *P*-values obtained via two-way ANOVA adjusted based on Bonferroni's multiple comparison test (**a**, **c**, **d**) or an unpaired Student's *t*-test (**b**) (*$P < 0.05$; **$P < 0.01$;****$P < 0.001$). Exact *P*-values are indicated in the Source Data file.

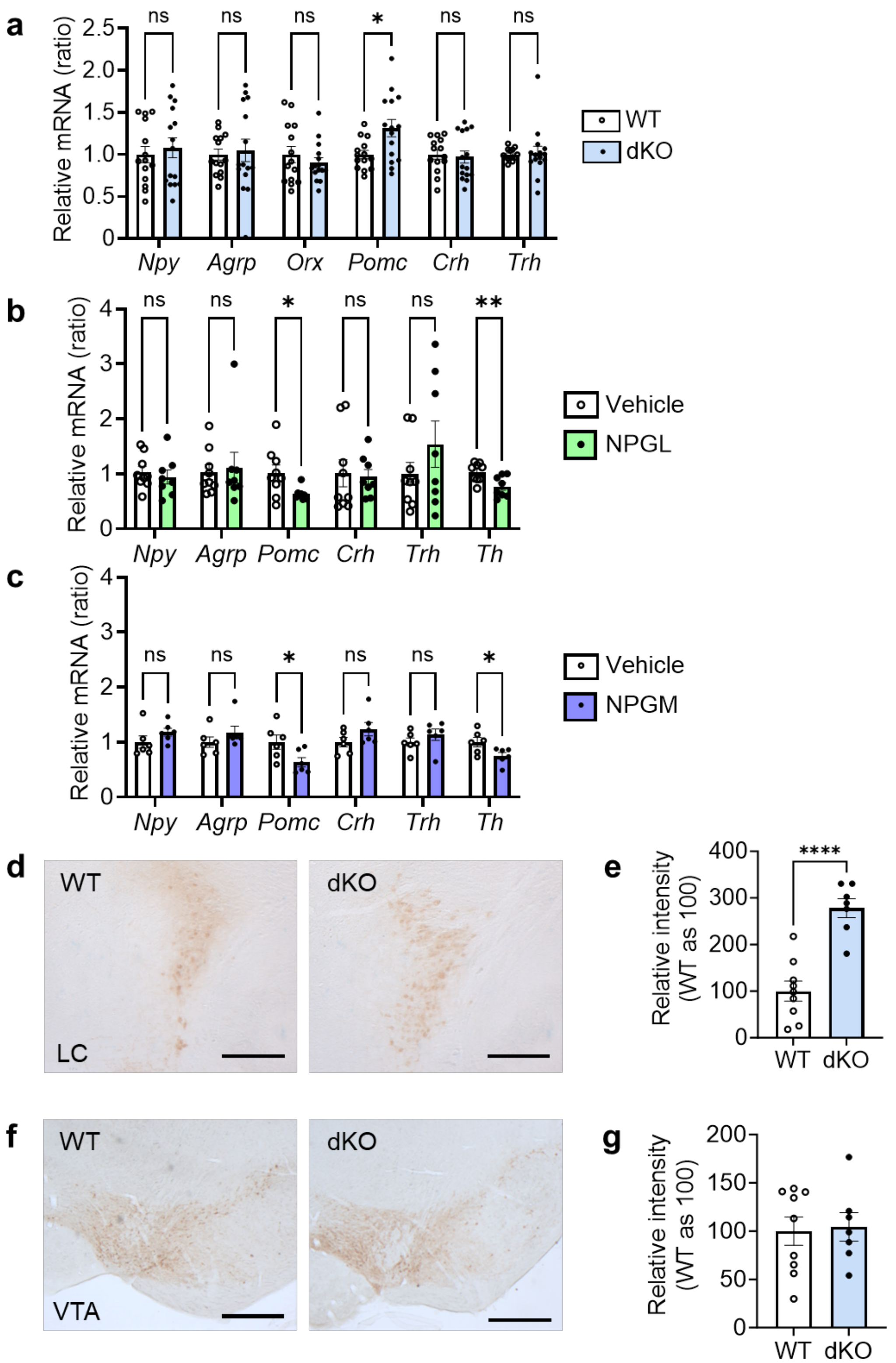

Supplementary Figure 6. Regulation of neural factor by the NPGL/NPGM system.

a–c, quantitative PCR (qPCR) analysis in mediobasal hypothalmus. **a**, Wild-type (WT) or *Npgl*- and *Npgm*-double knockout (dKO) male mice fed a normal diet (ND). Relative mRNA expression

levels of hypothalamic neuropeptides associate with feeding behavior (*neuropeptide Y*, *Npy*; *agouti-related protein*, *Agrp*; *orexin*, *Orx*; *pro-opiomelanocortin*, *Pomc*; *corticotropin-releasing hormone*, *Crh*; *thyrotropin-releasing hormone*, *Trh*) (n = 14 for WT and n = 15 for dKO). **b**, **c**, Effect of intracerebroventricular (ICV) injection of NPGL or NPGM (1 nmol/mouse) on the levels of mRNA associated with feeding regulation in WT mice. Relative mRNA expression of *Npy*, *Agrp*, *Pomc*, *Crh*, *Trh*, and *tyrosine hydroxylase* (*Th*) genes after NPGL (n = 9 for vehicle and n = 8 for NPGL) (**b**) or NPGM (n = 6) (**c**) injection. **d**–**g**, Representative immunohistochemistry images of TH protein expression in the locus coeruleus (LC) (**d**) or substantia nigra pars compacta (SNc) and ventral tegmental area (VTA) (**f**) of the WT and dKO mice fed a ND. Relative intensity of TH protein staining in the LC (**e**) or SNc and VTA (**g**) (n = 9 for WT and n = 7 for dKO mice). Data are presented as the mean ± SEM. For bar graphs, each dot represents data from an individual mouse. Asterisks refer to *P*-values obtained from unpaired Student's *t*-tests (**a**, **b**, **c**, **e**, **g**) (*$P < 0.05$; **$P < 0.01$; ****$P < 0.001$; ns: not significant, $P > 0.05$). Exact *P*-values are indicated in the Source Data file. Scale bars: 100 μm (**d**, **f**).

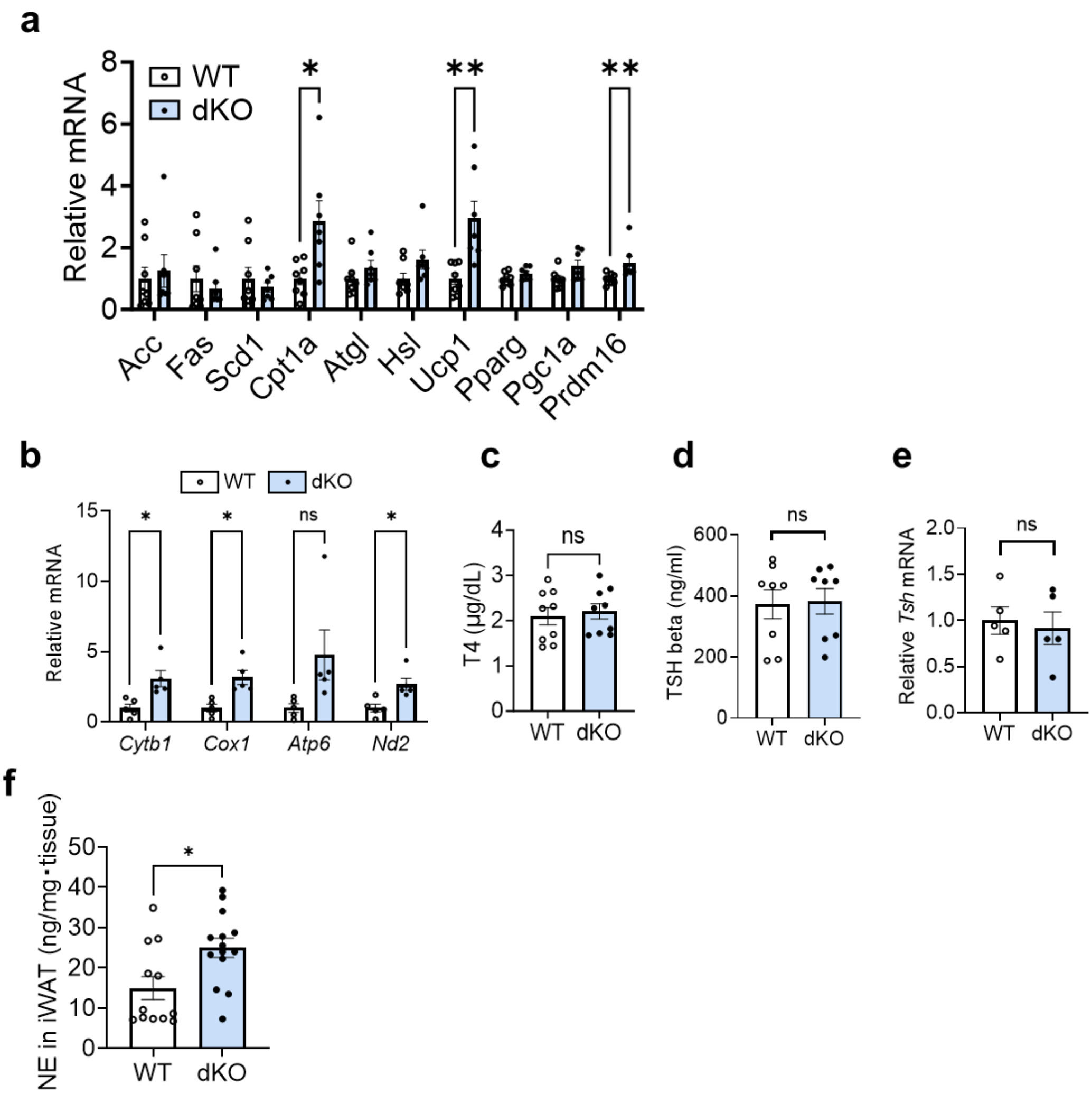

Supplementary Figure 7. Deletion of the NPGL/NPGM system affects thermogenesis independently of thyroid hormone.

Wild-type (WT) or *Npgl*- and *Npgm*-double knockout (dKO) male mice were fed a normal diet (ND). **a**, Relative mRNA expression levels related to lipogenesis (*acetyl-CoA carboxylase*, *Acc*; *fatty acid synthase*, *Fas*; *stearoyl-CoA desaturase 1*, *Scd1*), lipolysis (*carnitine palmitoyltransferase 1a*, *Cpt1a*; *adipose triglyceride lipase*, *Atgl*; *hormone-sensitive lipase*, *Hsl*), thermogenesis (*uncoupling protein 1*, *Ucp1*), and differentiation (*peroxisome proliferator-activated receptor γ*, *Pparg*; *peroxisome proliferator-activated receptor γ co-activator-1 α*, *Pgc1a*; *PRD1-BF-1-RIZ1 homologous domain containing protein 16*, *Prdm16*) in the brown adipose tissue (BAT) of mice (n = 8 for WT and n =7 for dKO mice). **b**, Relative levels of mitochondrial mRNA related to oxidative phosphorylation (*cytochrome b*, *Cytb1*; *cytochrome oxidase 1*, *Cox1*; *ATP synthase subunit 6*, *Atp6*; *NADH dehydrogenase 2*, *Nd2*) in the BAT of mice fed an ND (n = 5). **c**, Serum free T4 levels in WT and dKO mice (n = 9). **d**, Serum thyroid stimulating hormone subunit beta (TSH-beta) levels in WT and dKO mice at 18 weeks of age (n = 8). **e**, Relative mRNA

expression of TSH-beta levels in pituitary of WT and dKO mice (n = 5). **f,** Norepinephrine (NE) content in inguinal white adipose tissue (iWAT) (n = 12 for WT and n = 14 for dKO mice). Data are presented as the mean ± SEM. For bar graphs, each dot represents data from an individual mouse. Asterisks refer to *P*-values obtained from unpaired Student's *t*-tests (**a–f**) (*$P < 0.05$; **$P < 0.01$; ns: not significant, $P > 0.05$). Exact *P*-values are indicated in the Source Data file.

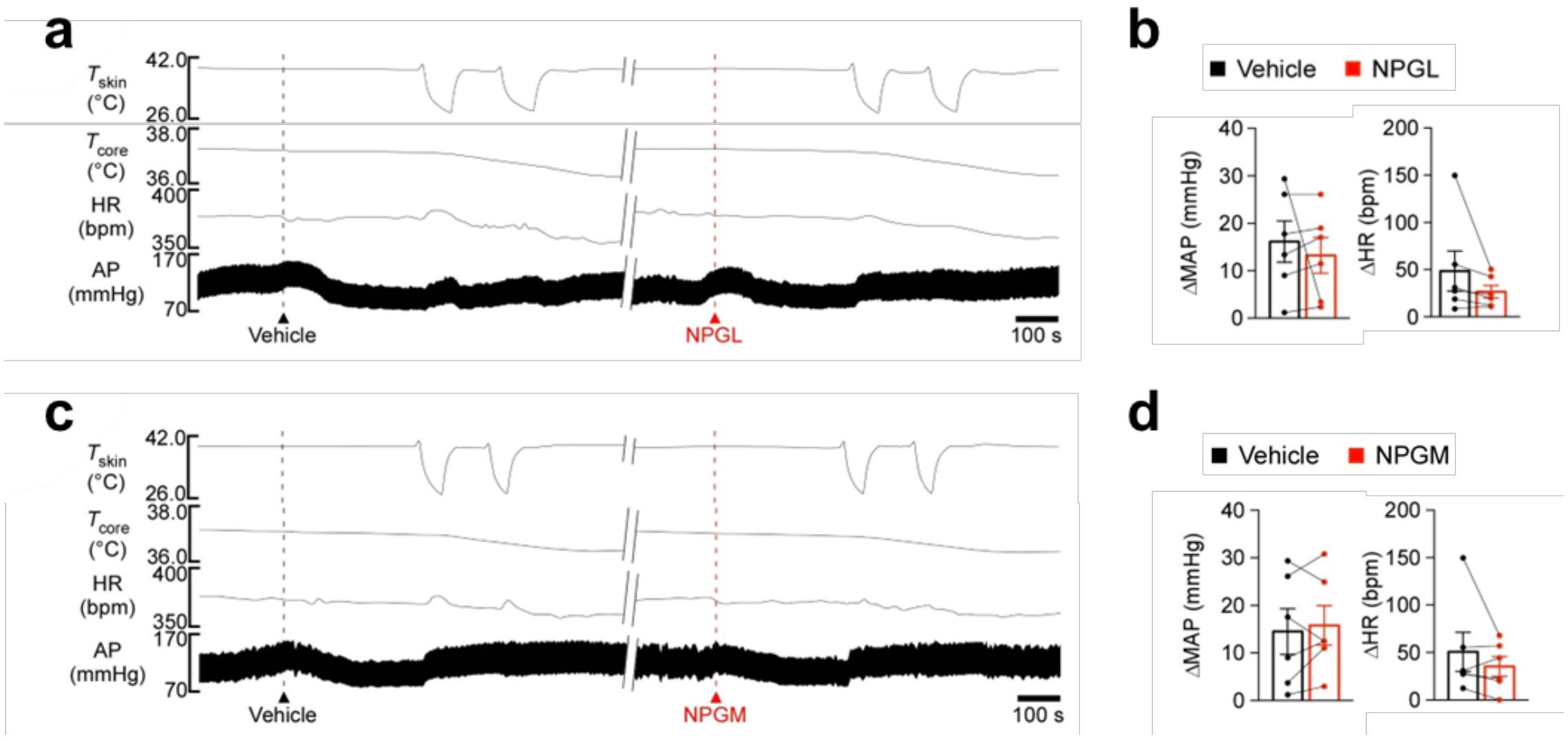

Supplementary Figure 8. Central actions of NPGL/NPGM do not affect the cardiovascular system.

Effects of NPGL and NPGM intracerebroventricular (ICV) injections on skin cooling-evoked physiological responses. Changes in core temperature (T_{core}), heart rate (HR), and arterial pressure (AP) after vehicle, NPGL (**a**, **b**) (n = 6) and NPGM injections (**c**, **d**) (n = 6) are shown. Skin cooling-evoked changes in the physiological variables were quantitatively analyzed. Skin temperature (T_{skin}) was monitored to indicate the timing and magnitude of the cooling stimulus. The waveform data shown here correspond to the same experiments presented in Fig. 3i,k. Data are presented as the mean ± SEM. In the bar graphs, each dot represents data from an individual mouse. No significant differences were observed between the vehicle and NPGL groups (**b**, **d**; paired *t*-test, $P > 0.05$). Exact *P*-values are provided in the Source Data file.

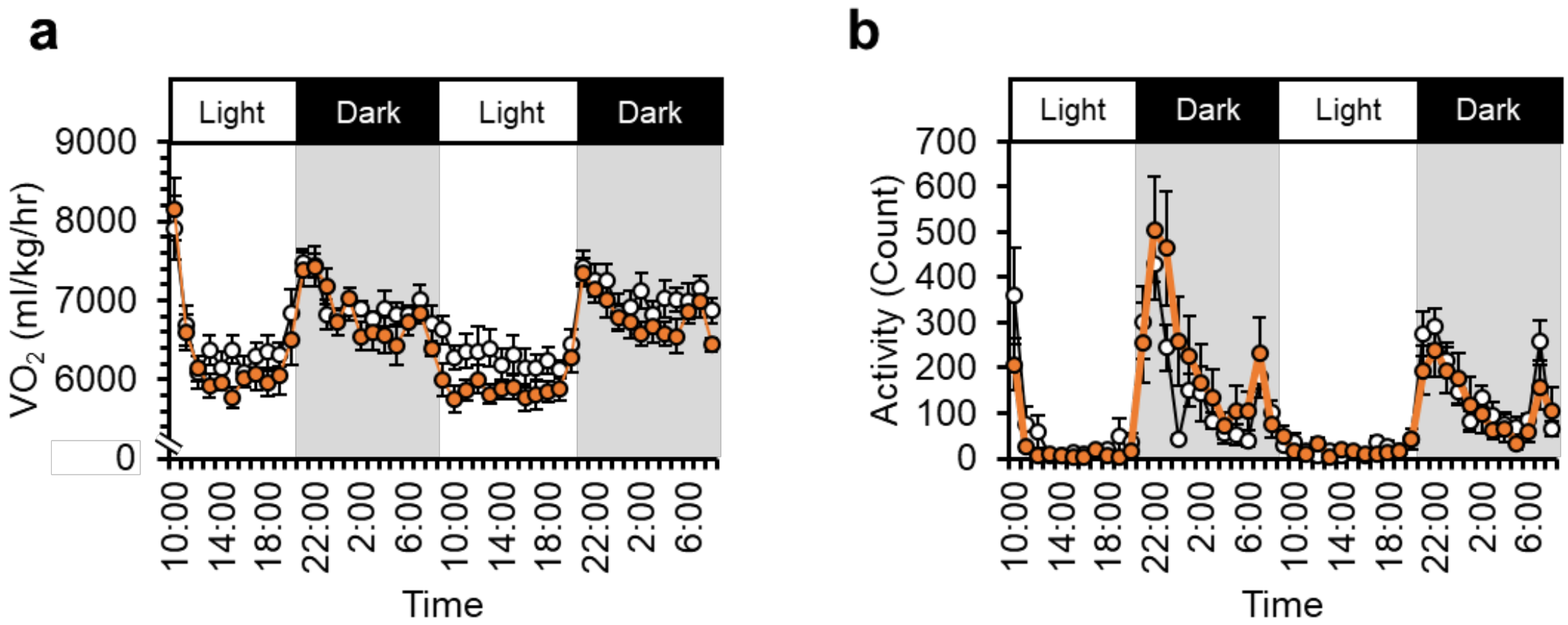

Supplementary Figure 9. Silencing of *Npgl* and *Npgm* genes.

Silencing of *Npgl* and *Npgm* using small interfering RNA (siRNA) in wild-type mice fed a high-fat diet. **a**, **b**, Whole-body oxygen consumption (VO_2) (**a**) and locomotor activity (**b**) were measured within one to two weeks after injection (n = 8). Data are presented as the mean ± SEM. *P*-values were calculated using two-way ANOVA adjusted based on Bonferroni's multiple comparison test (**a**, **b**). Exact *P*-values are indicated in the Source Data file.

Supplementary Table 1 | gRNAs for CRISPR Cas9 system and genotype primers

gRNA sequences

Npgl-1 (61-38)	ACAGGGAGCAGGCGAGACTCAGG
Npgl-2 (380-402)	AAGTGCTGACGAGACATTCAGGG
Npgm-1 (58-36)	TGAACAGCAGAACGCAGCCCAGG
Npgm-2 (444-466)	CCACTTAGTTCGATTATTTCAGG

Genotype primers

Npgl forward (388-408)	CAGAAGCAGGATCCACAGCTC
Npgl reverse (1184-1164)	TCCCCACTCTCAGCTCCTAT
Npgm forward ((-178)-(-159))	CAGCTGGTTGGCAAATTGGA
Npgm reverse (631-610)	TCCTATGAACTACACATGCGGT

Supplementary Table 2 | qPCR primers

Gene	Forward (5'→3')	Reverse (5'→3')
Npgl	CAAATGCAAAAGCGGGGCT	CAGTGGGGTGGTCAATCAGT
Npgm	AACTCCTTCATCCCGGGTCT	CAGCGTCAGAGAGCAAGTGT
Npy	TATCTCTGCTCGTGTGTTTG	GATTGATGTAGTGTCGCAGA
Agrp	TGTTCCCAGAGTTCCCAGGTC	GCATTGAAGAAGCGGCAGTAGCAC
Pomc	ACGTCCAAACCCTCGTTTCT	TTCCACGTGTCAGGCCTAGT
Crh	GGCATCCTGAGAGAAGTCCC	TGTTAGGGGCGCTCTCTTCT
Trh	TTGGTGCTGCCTTAGATTCCT	GGCTCCCACTTCTCCCAAAT
Th	TACAAGCAGGGTGAGCCAAT	GCTGTGGAATGCTGTCCTCT
Tsh	GGAGAGTGGGTCATCACAGC	GCAGCACTCATGCTCTCCA
Acc	TCCGCACTGACTGTAACCACAT	TGCTCCGCACAGATTCTTCA
Fas	AGGGGTCGACCTGGTCCTCA	GCCATGCCCAGAGGGTGGTT
Scd1	CTGTACGGGATCATACTGGTTC	GCCGTGCCTTGTAAGTTCTG
Cpt1a	CCTGGGCATGATTGCAAAG	GGACGCCACTCACGATGTT
Atgl	AACACCAGCATCCAGTTCAA	GGTTCAGTAGGCCATTCCTC
Hsl	GCTGGGCTGTCAAGCACTGT	GTAACTGGGTAGGCTGCCAT
Ucp1	CAAAAACAGAAGGATTGCCGAAA	TCTTGGACTGAGTCGTAGAGG
Pparg	CAGGCTTGCTGAACGTGAAG	GGAGCACCTTGGCGAACA
Pgc1a	CCCTGCCATTGTTAAGACC	TGCTGCTGTTCCTGTTTTC
Prdm16	CAGCACGGTGAAGCCATTC	GCGTGCATCCGCTTGTG
Cytb1	CCTTCATGTCGGACGAGGCTT	TGCTGTGGCTATGACTGCGAA
Cox1	TAGCCCATGCAGGAGCATCA	TGGCTGGGGGTTTCATGTTGA
Atp6	TGGCATTAGCAGTCCGGCTT	ATGGTAGCTGTTGGTGGGCT
Nd2	GCCTGGAATTCAGCCTACTAGC	GGCTGTTGCTTGTGTGACGA
Actb	GGCACCACACCTTCTACAAT	AGGTCTCAAACATGATCTGG

Supplementary Table 3 | siRNA sequences

**Target**	**Sequence 5'→3'(sense)**	**Sequence 5'→3'(antisense)**
si-Control	AAGGCUGACAUGAUUCUGAdTdT	UCAGAAUCAUGUCAGCCUUdTdT
si-NPGL	GAAGUCAUCUGAGAACUUGdTdT	CAAGUUCUCAGAUGACUUCdTdT
si-NPGM	GCGUUCUGCUGUUCAGUCUdTdT	AGACUGAACAGCAGAACGCdTdT

Supplementary Table 4 | Antibody's list

**Name of primary antibody**	**Company**	**Cat. Number**	**Mono/Polyclonal**	**Source**	**Dilution for WB**	**Dilution for IHC**
Tyrosine hydroxyllase (TH)	Sigma-Aldrich	AB152	Poly	Rabbit	1:1000	1:1000
UCP1	Cell Signalling Technology	14670S	Poly	Rabbit	1:1000	—
Beta-actin (ACTB)	Sigma-Aldrich	A3854	Poly	Rabbit	1:10000	—
NPGL	Provided by Prof. Ukena		Poly	Rabbit	—	1:250
NPGM	Provided by Prof. Ukena		Poly	Guinea Pig	—	1:250
Tyrosine hydroxyllase (TH)	Merck Millipore	MAB318	Mono	Mouse	—	1:1000

**Name of secondary antibody**	**Company**	**Cat. Number**	**Mono/Polyclonal**	**Source**	**Dilution for WB**	**Dilution for IHC**
HRP-conjugated anti-Rabbit IgG	Jackson Immuno Research	AB2313567	Poly	Goat	1:10000	—
Biotinylated anti-Rabbit IgG	Vectro Laboratories	PK-4001	Poly	Goat	—	1:1000
Alexa Fluor 488 anti-rabbit IgG	Invitrogen	A21206	Poly	Donkey	—	1:500
Alexa Fluor 594 anti-mouse IgG	Invitrogen	A21203	Poly	Donkey	—	1:500
Alexa Fluor 594 anti-guinea pig IgG	Invitrogen	A11076	Poly	Donkey	—	1:500
