# Supplementary material for: Deletion of neurosecretory proteins GL and GM drives dual anti-obesity effects via appetite suppression and enhanced energy expenditure
Source: Commun Biol. 2026 Jun 3;9:1168. doi: 10.1038/s42003-026-10405-7 (PMC13538515; doi:10.1038/s42003-026-10405-7)
Supplement: Supplementary file 2 [file 42003_2026_10405_MOESM2_ESM.pdf]

Description of Additional Supplementary Files

File name: Supplementary Data 1

Description: Source data underlying the graphs and charts presented in the main figures.
