# Supplementary material for: Deletion of neurosecretory proteins GL and GM drives dual anti-obesity effects via appetite suppression and enhanced energy expenditure
Source: Commun Biol. 2026 Jun 3;9:1168. doi: 10.1038/s42003-026-10405-7 (PMC13538515; doi:10.1038/s42003-026-10405-7)
Supplement: Supplementary file 3 [file 42003_2026_10405_MOESM4_ESM.pdf]

nature portfolio

Corresponding author(s): Reiko Hanada

Last updated by author(s): Apr 23, 2026

Reporting Summary

Nature Portfolio wishes to improve the reproducibility of the work that we publish. This form provides structure for consistency and transparency in reporting. For further information on Nature Portfolio policies, see our Editorial Policies and the Editorial Policy Checklist.

Statistics

For all statistical analyses, confirm that the following items are present in the figure legend, table legend, main text, or Methods section.

n/a	Confirmed	
☐	☒	The exact sample size (*n*) for each experimental group/condition, given as a discrete number and unit of measurement
☐	☒	A statement on whether measurements were taken from distinct samples or whether the same sample was measured repeatedly
☐	☒	The statistical test(s) used AND whether they are one- or two-sided<br>*Only common tests should be described solely by name; describe more complex techniques in the Methods section.*
☒	☐	A description of all covariates tested
☐	☒	A description of any assumptions or corrections, such as tests of normality and adjustment for multiple comparisons
☐	☒	A full description of the statistical parameters including central tendency (e.g. means) or other basic estimates (e.g. regression coefficient) AND variation (e.g. standard deviation) or associated estimates of uncertainty (e.g. confidence intervals)
☐	☒	For null hypothesis testing, the test statistic (e.g. *F*, *t*, *r*) with confidence intervals, effect sizes, degrees of freedom and *P* value noted<br>*Give P values as exact values whenever suitable.*
☒	☐	For Bayesian analysis, information on the choice of priors and Markov chain Monte Carlo settings
☒	☐	For hierarchical and complex designs, identification of the appropriate level for tests and full reporting of outcomes
☒	☐	Estimates of effect sizes (e.g. Cohen's *d*, Pearson's *r*), indicating how they were calculated

Our web collection on statistics for biologists contains articles on many of the points above.

Software and code

Policy information about availability of computer code

Data collection	µCT analysis was recorded by SkyScan 1276 X-ray µCT system (Bruker). O2 consumptions and CO2 production were measured using an Oxymax-CLAMS system (Columbus Instruments) or a capnometer (OLG-2800; Nihon Kohden). Western blot were recroded by Fusion SOLO S (Vilber). LC-MS/MS analysis were performed using LCMS-8040 spectrometer (Shimadzu Corporation). RT-qPCR was using LightCycler 96 system (Roche). Hematoxylin and eosin (HE) staining, immunostaining, and TEM were observed using Axio Imager M2 microscope (Carl Zeiss), confocal microscope (LSM710 or LSM900; Carl Zeiss), and transmission electron microscope (H-7650; Hitachi).<br>Online resource, databasese used:<br>Ensembl Biomart (https://grch37.ensembl.org/biomart/martview/, release110,GRCh37)<br>Ensembl (https://grch37.ensembl.org/Tools/VEP)<br>Open Targets (https://platform.opentargets.org/downloads, version:23.09)
Data analysis	ImageJ (image processing), GraphPad Prism 9 and 10 (graph and statistical analysis)

For manuscripts utilizing custom algorithms or software that are central to the research but not yet described in published literature, software must be made available to editors and reviewers. We strongly encourage code deposition in a community repository (e.g. GitHub). See the Nature Portfolio guidelines for submitting code & software for further information.

Data

Policy information about availability of data

All manuscripts must include a data availability statement. This statement should provide the following information, where applicable:

- Accession codes, unique identifiers, or web links for publicly available datasets
- A description of any restrictions on data availability
- For clinical datasets or third party data, please ensure that the statement adheres to our policy

Data generated or analyzed in the present study are included in this published article and its supplementary information files. Further information and requests should be directed to and will be fulfilled by the corresponding author.

Research involving human participants, their data, or biological material

Policy information about studies with human participants or human data. See also policy information about sex, gender (identity/presentation), and sexual orientation and race, ethnicity and racism.

Reporting on sex and gender	Not applicable
Reporting on race, ethnicity, or other socially relevant groupings	Not applicable
Population characteristics	Not applicable
Recruitment	Not applicable
Ethics oversight	Not applicable

Note that full information on the approval of the study protocol must also be provided in the manuscript.

Field-specific reporting

Please select the one below that is the best fit for your research. If you are not sure, read the appropriate sections before making your selection.

☒ Life sciences ☐ Behavioural & social sciences ☐ Ecological, evolutionary & environmental sciences

For a reference copy of the document with all sections, see nature.com/documents/nr-reporting-summary-flat.pdf

Life sciences study design

All studies must disclose on these points even when the disclosure is negative.

Sample size	Sample sizes were determined to ensure the reproducibility and reliability of the study outcome according to a pilot study as well as the basis previous experimental experience.
Data exclusions	We did not exclude any data from the analysis.
Replication	Each experiment was performed with at least 4 biological replicates. All the reported results are from experiments in which each repeat gave similar results.
Randomization	All samples were randomly allocated into experimental groups.
Blinding	No formal blinding was used.

Reporting for specific materials, systems and methods

We require information from authors about some types of materials, experimental systems and methods used in many studies. Here, indicate whether each material, system or method listed is relevant to your study. If you are not sure if a list item applies to your research, read the appropriate section before selecting a response.

Materials & experimental systems

n/a	Involved in the study
	☒ Antibodies
☒	☐ Eukaryotic cell lines
☒	☐ Palaeontology and archaeology
	☒ Animals and other organisms
☒	☐ Clinical data
☒	☐ Dual use research of concern
☒	☐ Plants

Methods

n/a	Involved in the study
☒	☐ ChIP-seq
☒	☐ Flow cytometry
☒	☐ MRI-based neuroimaging

Antibodies

Antibodies used

Rabbit anti-mouse TH antibody (Cat# AB152, Lot# 3814879, Sigma-Aldrich), Rabbit anti-mouse UCP1 antibody (Cat#14670S, Lot# 1, Cell Signalling Technology), Rabbit anti-mouse β-actin antibody (Cat# A3854, Lot# 015M4866V, Clone name AC-15, Sigma-Aldrich), goat anti-rabbit IgG antibody (Cat# 111-035-003, Lot# 100103, RRID AB2313567, Jackson ImmunoResearch), biotinylated goat anti-rabbit IgG (Cat# PK-4001, Lot# ZF0606, Vector Laboratories), mouse anti-mouse TH antibody (Cat#MAB318, Lot# 3506431, Clone name: LNC1 Merck Millipore) Alexa Fluor 488 conjugate donkey anti-rabbit IgG (Cat# A21206, Lot# 2541645, Invitrogen), Alexa Fluor 594 conjugate donkey anti-mouse IgG (Cat# A21203,Lot# 987237, Invitrogen), Alexa Fluor 594 conjugate donkey anti-guinea pig IgG (Cat# A11076, Lot# 556434, Invitrogen).
Rabbit anti-mouse NPGL antibody and Guinea pig anti-mouse NPGM antibody provided by Prof. Ukena.

Validation

All antibodies used in this study have been validated by their respective manufacturer's . Additional testing was performed in the laboratory by titrating on mouse samples per the manufacturer's recommendations.

Animals and other research organisms

Policy information about studies involving animals; ARRIVE guidelines recommended for reporting animal research, and Sex and Gender in Research

Laboratory animals

Mouse wild type (C57BL/6J and ICR , SLC Japan and CLEA Japan), rat wild type (Slc: Wistar/ST, SLC Japan)
Npgl and Npgm knockout mice were generated in our laboratory using CRISPR-Cas9 system (Integrated DNA Technologies, Coralville)

Wild animals

This study did not involve field-collected samples.

Reporting on sex

In experiments with mice and rats, male were used.

Field-collected samples

This study did not involve field-collected samples.

Ethics oversight

All experimental procedures were performed according to institutional and national guidelines and regulations. The study protocol was approved by Oita University (approval number 170302, 230301, 30-5, and 2-18) and Nagoya University (approval number #M220097-002).

Note that full information on the approval of the study protocol must also be provided in the manuscript.

Plants

Seed stocks

Not applicable

Novel plant genotypes

Not applicable

Authentication

Not applicable
